# In Vitro Effects of a Small-Molecule Antagonist of the Tcf/ß-Catenin Complex on Endometrial and Endometriotic Cells of Patients with Endometriosis

**DOI:** 10.1371/journal.pone.0061690

**Published:** 2013-04-23

**Authors:** Sachiko Matsuzaki, Claude Darcha

**Affiliations:** 1 CHU Clermont-Ferrand, CHU Estaing, Chirurgie Gynécologique, 1, Clermont-Ferrand, France; 2 Clermont Université, Université d’Auvergne, ISIT UMR6284, Clermont-Ferrand, France; 3 CNRS, ISIT UMR6284, Clermont-Ferrand, France; 4 CHU Clermont-Ferrand, Service d’Anatomie et Cytologie Pathologiques, Clermont-Ferrand, France; University of Kentucky, United States of America

## Abstract

**Background:**

Our previous studies suggested that aberrant activation of Wnt/ß-catenin signaling might be involved in the pathophysiology of endometriosis. We hypothesized that inhibition of Wnt/ß-catenin signaling might result in inhibition of cell proliferation, migration, and/or invasion of endometrial and endometriotic epithelial and stromal cells of patients with endometriosis.

**Objectives:**

The aim of the present study was to evaluate the effects of a small-molecule antagonist of the Tcf/ß-catenin complex (PKF 115–584) on cell proliferation, migration, and invasion of endometrial and endometriotic epithelial and stromal cells.

**Methods:**

One hundred twenty-six patients (78 with and 48 without endometriosis) with normal menstrual cycles were recruited. In vitro effects of PKF 115–584 on cell proliferation, migration, and invasion and on the Tcf/ß-catenin target genes were evaluated in endometrial epithelial and stromal cells of patients with and without endometriosis, and in endometrial and endometriotic epithelial and stromal cells of the same patients.

**Results:**

The inhibitory effects of PKF 115–584 on cell migration and invasion in endometrial epithelial and stromal cells of patients with endometriosis prepared from the menstrual phase were significantly higher than those of patients without endometriosis. Levels of total and active forms of MMP-9 were significantly higher in epithelial and stromal cells prepared from menstrual endometrium in patients with endometriosis compared to patients without endometriosis. Treatment with PKF 115–584 inhibited MMP-9 activity to undetectable levels in both menstrual endometrial epithelial and stromal cells of patients with endometriosis. The number of invasive cells was significantly higher in epithelial and stromal cells of endometriotic tissue compared with matched eutopic endometrium of the same patients. Treatment with PKF 115–584 decreased the number of invasive endometriotic epithelial cells by 73% and stromal cells by 75%.

**Conclusions:**

The present findings demonstrated that cellular mechanisms known to be involved in endometriotic lesion development are inhibited by targeting the Wnt/β-catenin pathway.

## Introduction

Endometriosis, a common cause of infertility and pelvic pain, is defined as the presence of endometrial glands and stroma within extra-uterine sites [1]. Endometriosis affects approximately 10% of women of reproductive age [1]. However, despite extensive studies, its etiology, pathogenesis, and pathophysiology are not fully understood. Knowledge of these factors is indispensable for the development of strategies for prevention and targeted treatment of endometriosis.

Our previous study suggested that the Wnt/ß-catenin signaling pathway may be aberrantly activated in the endometrium of patients with endometriosis during the mid-secretory phase [2], as well as in endometriotic tissues [3]. The Wnt/ß-catenin pathway is involved in development, tissue self-renewal, and various diseases [4]–[7]. In the absence of Wnt-initiated signal (“off” state), ß-catenin is targeted for degradation by the APC/Axin/GSK-3ß complex [4]–[7]. Binding of Wnt ligands to a Frizzled/LRP receptor complex (“on” state) leads to the inactivation of GSK-3ß and accumulation of cytosolic ß-catenin, which then translocates into the nucleus, where it binds to Tcf/LEF transcription factors to activate transcription of Wnt-responsive genes such as those involved in cell proliferation, migration, and invasion [4]–[7]. These processes are also involved in the pathophysiology of endometriosis [1]. We hypothesized that if aberrant activation of Wnt/ß-catenin is involved in the pathophysiology of endometriosis, inhibition of this signaling might result in reduced cell proliferation, migration, and/or invasion of endometriotic and endometrial cells of patients with endometriosis.

To date, a number of components have been identified that target different steps in the Wnt/ß-catenin pathway [4]–[13]. Of these steps, a promising drug target may be the critical protein-protein interaction between ß-catenin and Tcf. Several small-molecule antagonists of the Tcf/ß-catenin complex disrupt this critical protein-protein interaction [14]. Of these, two fungal derivatives (PKF 115–854 and CGP049090) fulfill nearly every tested prediction, including disruption of Tcf/ß-catenin complexes in vitro and inhibition of colon cancer cell proliferation, ß-catenin–responsive transcription, and ß-catenin–mediated axis duplication in *Xenopus* embryos [9].

The objective of the present study was to evaluate the effects of small-molecule antagonists of the Tcf/ß-catenin complex (PKF 115–584 and CGP049090) on cell proliferation, migration, and invasion of endometrial and endometriotic epithelial and stromal cells obtained from patients with and without endometriosis (controls) throughout the menstrual cycle.

## Materials and Methods

### Ethics Statement

The research protocol was approved by the Consultative Committee for Protection of Persons in Biomedical Research (CCPPRB) of the Auvergne (France) region. Informed written consent was obtained from each patient prior to tissue collection.

### Patients

Patients age 20–37 years undergoing laparoscopy for endometriosis were recruited at CHU Clermont-Ferrand for the present study. As control samples, endometrial tissues were obtained from patients with uterine myomas who underwent laparoscopic myomectomy or patients who underwent laparoscopic surgery for tubal infertility. None of the women had received hormonal treatments, such as gonadotropin-releasing hormone agonists (GnRHa) or sex steroids, and none used intrauterine contraception for at least 6 months prior to surgery.

Recruited patients had regular menstrual cycles (26–32 days) with confirmation of their menstrual history. Published endometrial dating criteria [15] and menstrual history were utilized to assess the menstrual cycle phase. Endometrial dating was performed independently by C.D. and an independent pathologist. All patients, independent of group, were selected for the present study based on consistent histological findings and menstrual history. Endometrial biopsies were classified into one of five groups: proliferative (P) (days 8–14), early-secretory (ES) (days 15–19), mid-secretory (MS) (days 20–24), late-secretory (LS) (days 25–28) [21], [22] and menstrual (days 1–3).

Samples from 78 patients who had histological evidence of pelvic endometriosis and samples from 30 patients with uterine myomas or samples from 18 patients of tubal infertility were used for the present analysis. Samples of tissue representing deep endometriotic lesions, ovarian endometriosis, or superficial peritoneal endometriosis (ectopic endometrium) were paired with eutopic endometrial samples of the same patient for analyses. Laparoscopic surgical treatment for endometriosis was performed with a four-puncture technique [16]. Deep infiltrating endometriosis was completely excised using mechanical instruments and electrosurgery. Ovarian endometriomas were excised by the previously described stripping technique [16]. Peritoneal superficial endometriotic lesions were excised using a pair of scissors without coagulation.

Deep infiltrating endometriosis was defined as endometriosis located 5 mm under the peritoneal surface. Patients with endometriotic ovarian cysts >3 cm in diameter were also included. Superficial peritoneal endometriosis was defined as endometriosis located on the peritoneal surface.

Patients in which myomas had distorted the endometrial cavity were excluded. All of the patients with myomas in the present study had intramural and/or subserosal myomas. All of the patients with uterine myomas or tubal infertility had no endometriosis. The clinical characteristics of patients are shown in Table 1.

**Table 1:** Clinical characteristics of patients.

	Endometriosis			Uterine fibroma	Tubal infertility
	DE	OE	SE		
No of cases	33	27	18	30	18
Age^a^	31.0	30.5	31.0	30.5	30.0
	(21–37)	(21–37)	(20–37)	(22–37)	(21–37)
Parity^a^	0 (0–1)	0 (0–1)	0 (0–1)	0 (0–1)	0 (0–1)
rASRM stage^b^					
I	12	0	10		
II	6	0	8		
III	6	17	0		
IV	9	10	0		

a Median (range).

b Revised American Society for Reproductive Medicine classification (rASRM) (American Society for Reproductive Medicine, 1997).

DE: patients with deep infiltrating endometriosis.

OE: patients with ovarian endometriosis.

SE: patients with only superficial peritoneal endometriosis.

Endometrial tissue biopsies were performed just prior to surgery using an endometrial suction catheter (Pipelle, Laboratoire CCD, Paris, France). Samples of endometrial and endometriotic tissue were divided into two portions. The first tissue portion was fixed in 10% formalin-acetic acid and embedded in paraffin. The second portion was immediately collected in Hanks’ balanced salt solution (Life Technologies, Cergy Pontoise, France).

### Study Design

The present study first investigated whether small interfering RNA (siRNA)-mediated knockdown of ß-catenin, a key component of the Wnt signaling pathway, could inhibit expression of the Tcf/ß-catenin target genes Cyclin D1, c-Myc, and Survivin, as well as cell proliferation, in endometrial and endometriotic epithelial and stromal cells. Next, small-molecule antagonists of the Tcf/ß-catenin complex (PKF 115–584 and CGP049090) were evaluated for their ability to inhibit cell proliferation in endometrial and endometriotic epithelial and stromal cells. Finally, the effects of PKF 115–584 on cell proliferation, migration, and invasion, as well as on expression of the Tcf/ß-catenin target genes Cyclin D1, c-Myc, Survivin, MMP-2, and MMP-9, in endometrial and endometriotic epithelial and stromal cells were evaluated.

### Cell Culture

The endometrial and endometriotic tissues were carefully dissected and minced into 1–2 mm^3^ fragments incubated in phenol red-free DMEM/F-12 containing type I collagenase (0.25%) (Life Technologies) and deoxynuclease I (15 U/mL) (Life Technologies) for 60 min (endometrium) or 90 min (endometriosis) at 37°C. Endometrial or endometriotic cells were then separated by filtration through a 40-µm nylon cell strainer (BD, Le Pont de Claix, France). Epithelial cells that remained intact were retained by the strainer, whereas dispersed stromal cells passed through the strainer into the filtrate. Red blood cells were removed by hypotonic lysis (NH_4_Cl, 0.15 mol/L; KHCO_3_, 1 mmol/L; Na_2_ EDTA, 0.1 mmol/L) (Life Technologies). Isolated cells were plated onto Primaria flasks (BD) in phenol red-free DMEM/F-12 containing 10% charcoal-stripped FBS, 100 U/mL penicillin, 0.1 mg/mL streptomycin, and 0.25 µg/mL amphotericin B (Life Technologies) and incubated at 37°C in 95% air/5% CO_2_. Epithelial cells were incubated at 37°C in 95% air/5% CO_2_ for 60 min to allow contaminated stromal cells to attach to the flask wall. The nonattached epithelial cells were recovered and cultured in the culture medium into Primaria flasks (BD). The cells reached confluence in 2–3 days, and the first passages were used for experiments. Immunofluorescent staining was performed to determine the purity of the isolated endometrial and endometriotic epithelial and stromal cells using monoclonal antibodies for human cytokeratin (MNF116, 1∶100, DAKO, Glostrup, Denmark), vimentin (V9, 1∶100, DAKO), factor VIII (1∶100, DAKO), and CD 45 (1∶100, DAKO) (Figure S1), as previously described [17].

### ß-catenin siRNA Transfection

Cells were seeded into 96-well plates (1×10^4^ cells per well) for cell proliferation analysis, 24-well plates (5×10^4^ cells per well) for quantitative real-time RT-PCR, or 60-mm dishes (2×10^5^ cells per dish) for western blotting in culture media 24 h before transfection. siRNA transfections were performed in serum-free OPTI-MEM using 20 nM siRNAs and Lipofectamine. Control siRNA (AM4611, Life Technologies) or validated human ß-catenin siRNAs (siRNA ID: s437; siRNA ID:42816, Life Technologies) were added to cells and incubated for 24 h for quantitative real-time RT-PCR or 48 h for cell proliferation assays and western blotting. Mock-transfected (i.e., no siRNA) cells were used as negative controls. Then, cell proliferation assay, quantitative real-time RT-PCR for ß-catenin, Cyclin D1, c-Myc, Survivin, and Hyaluronidase-2 (negative control), and western blotting for ß-catenin were performed.

### Cell Proliferation Assays

Cell proliferation assays were performed using the CellTiter 96® AQueous One Solution Cell Proliferation Assay (MTS) (Promega, Charbonnières-les-Bains, France). Briefly, cells were seeded into 96-well plates, at a density of 1×10^4^ cells per well in 100 µL culture media. These cells were cultured at 37°C for 2 days until confluence. Then, cells were incubated for 48 h with 100 µL culture media (2% charcoal-stripped FBS) containing PKF 115–584 (6.25 µM) (Sigma-Aldrich, Lyon, France), CGP049090 (6.25 µM) (Sigma-Aldrich), or vehicle only. PKF 115–584 or CGP049090 were dissolved in dimethyl sulfoxide (DMSO, 0.1%) (Life Technologies). We confirmed that DMSO (0.1%) used as a control did not affect cell proliferation in the present study.

During preliminary experiments, cells were incubated in various concentrations of PKF 115–584 (Sigma-Aldrich) or CGP049090 (Sigma-Aldrich) (0, 0.01, 0.05, 0.25, 1.25 and 6.25 µM) for 48 h. For both compounds, maximum inhibitory effects were obtained at 6.25 µM. Then, as a time-dependent experiment, cells were incubated for 24-, 48-, and 72-h periods with 100 µL culture media containing PKF 115–584 or CGP049090 at 6.25 µM. Maximum inhibitory effects were obtained after the 48-h and 72-h incubations for both compounds. Therefore, in the present study, the effects of these two compounds on cell proliferation were evaluated at 6.25 µM for 48 h.

CellTiter 96® AQueous One Solution Cell Proliferation Assay Reagent (Promega) was added in an equal volume (20 µL per well) to all wells, and cells were incubated for 3 h at 37°C. Absorbance was then read at 490 nm using a Multiskan microplate reader (Thermo Scientific, Illkirch, France). All experiments were performed in triplicate.

### In vitro Migration and Invasion Assays

In vitro migration and invasion assays were performed using uncoated or Matrigel-coated 24-well chambers/microfilters (BD), respectively. Briefly, after rehydration of the chambers, cells (5×10^4^ cells per chamber) in 500 µL phenol red-free DMEM/F12 without FBS (Life Technologies) were seeded onto the upper chamber. In the lower chamber, 750 µL phenol red-free DMEM/F12 plus 10% charcoal-stripped FBS (Life Technologies) were added. PKF 115–584 (6.25 µM) or vehicle only was then added into the upper chamber. Cell motility/migration was measured as the number of cells that migrated from a defined area of the uncoated microfilter through micropores in 24 h. Cell invasion was measured as the number of invasive cells from a defined area of the Matrigel-coated microfilter through micropores in 24 h. All experiments were performed in triplicate. The micropore filters were stained with toluidine blue, and the number of cells that migrated through filters was counted in the entire area of each filter. To count cell numbers objectively, a computerized image analysis system consisting of a light microscope (Leica, Lyon, France) (X20 objective, X10 ocular) and a color charge-coupling device camera (Sony, Paris, France) were utilized.

### RNA Extraction

Cells were seeded into 24-well plates at a density of 5×10^4^ cells per well in 500 µL culture media. These cells were cultured at 37°C for 2 days until confluence. Cells were then incubated for 48 h with 500 µL culture media with 2% charcoal-stripped FBS containing PKF 115–584 (6.25 µM) or vehicle only. Total RNA was extracted using the Qiagen RNeasy Mini Kit according to the manufacturer’s instructions (Qiagen, Courtaboef, France). Briefly, after aspirating culture media completely, cell were lysed directly in the cell-culture plates. Then, lysates were mixed with an equal volume of 70% ethanol, and total RNA was purified using RNeasy mini spin columns. The eluted total RNA was stored at –80°C until use. To eliminate potential genomic DNA contamination, RNA samples were treated with DNaseI (15 U; DNaseI, Qiagen) at room temperature for 15 min.

### Examination of RNA Yield and Integrity

RNA yield and integrity were analyzed using the RNA 6000 Pico kit and the Agilent Bioanalyzer 2100 (Agilent Technologies, Santa Clara, CA, USA). The RNA 6000 Pico kit allows determination of the integrity of very small amounts of RNA as well as estimation of the quantity of the isolated RNA, which has a linear range of 200–5,000 pg/µL. The RNA integrity number (RIN) value was >8.0 in all of the samples included in the present analysis using real-time RT-PCR [2], [18], [19].

### Quantitative Real-time RT-PCR

mRNA expression of the Tcf/ß-catenin target genes Cyclin D1, Survivin, c-Myc, MMP-2, and MMP-9 (www.stanford.edu/group/nusselab/cgi-bin/wnt/target_genes) as well as a non–Tcf/ß-catenin target gene, Hyaluronidase-2 (negative control), in non-treated and treated cells was measured by quantitative real-time RT-PCR with a Light Cycler as previously described [2], [20]. PCR amplification was performed using the FastStart DNA Master SYBR Green I kit (Roche, Mannheim, Germany). Primer sets are shown in Table S1. Quantification of the targets in the unknown samples was performed using a relative quantification method with external standards. The target concentration was expressed relative to the concentration of a reference housekeeping gene, glyceraldehyde 3-phosphate dehydrogenase (GAPDH). After each run, a melting curve analysis was performed to verify the specificity of the PCR reaction. The procedure was repeated independently three times to ensure the reproducibility of the results. All of the samples with a cycle threshold (Ct) coefficient of variation value >5% were retested.

### Western Blotting

Cells were seeded in triplicate at 2×10^5^ cells/60-mm dish in 3 mL culture media. These cells were cultured at 37°C for 2 days until confluence. Then, cells were incubated for 24 h with 3 mL culture media with 2% charcoal-stripped FBS containing PKF 115–584 (6.25 µM) or vehicle only. Cell lysates were isolated using M-PER Mammalian Protein Extraction Reagent (Thermo Scientific). Protein quantity in the cell lysates was evaluated by the Bradford protein assay following the manufacturer’s instructions (Bio-Rad Laboratories, Hercules, CA, USA). Twenty micrograms of total protein lysates were loaded onto 4–10% SDS-polyacrylamide gels and transferred to nitrocellulose membrane (GE Healthcare, Buckinghamshire, UK). Blots were processed as described in the SNAP i.d. Protein Detection System User Guide (Merck Millipore, Molsheim, France). Briefly, after the blot holders containing the blots were placed in the SNAP i.d. system, blocking buffer was added, and the vacuum was immediately activated. Primary antibodies for Cyclin D1 (Merck Millipore), ß-catenin (DAKO), and Actin (Sigma-Aldrich) were used. Primary antibodies diluted (Cyclin D1∶1:30; ß-catenin: 1∶800; Actin: 1∶500) in blocking buffer were added to the blot holders and incubated for 10 min at room temperature. The vacuum was initiated and the blots were washed three times with Tris-buffered saline Tween-20 (TBST). After the vacuum was turned off, the blots were incubated with horseradish peroxidase (HRP)-conjugated secondary antibodies goat anti-mouse IgG (Merck Millipore) for Cyclin D1 and ß-catenin and goat ant-rabbit IgG (Merck Millipore) for Actin diluted (1∶2500) in blocking buffer for an additional 10 min at room temperature. The vacuum was activated once again and the blots were washed three additional times with TBST prior to visualization of the immunoreactive proteins. Bound antibodies were detected using an ECL plus western blotting detection system (GE Healthcare) and exposure to x-ray film (GE Healthcare). Western blots were scanned and quantified by ImageJ software developed at the National Institute of Health.

### Total (pro- and Active Forms) and Active Forms of MMP-2 and MMP-9

Cells were seeded onto 24-well plates at a density of 1×10^5^ cells per well in 500 µL culture media. These cells were cultured at 37°C for 2 days until confluence. Cells were then incubated for another 24 h in culture media with 2% charcoal-stripped FBS containing PKF 115–584 (6.25 µM) or vehicle only. The supernatants from the cell culture were collected after 10 min centrifugation (1,000×g). Then, total and active forms of MMP-2 and MMP-9 were quantified in the culture supernatants using protein-specific Biotrak assay systems (MMP-2 Biotrak Activity Assay RPN 2631; MMP-9 Biotrak Activity Assay RPN2634, GE Healthcare) according to the manufacturer’s instructions. Absorbance was read at 405 nm using a Multiskan microplate reader (Thermo Scientific). Total and active forms of MMP-2 and MMP-9 were calculated using standards provided by the kit and expressed as ng/mL. The amount of total protein in the culture supernatants was measured using the Bio-Rad protein assay (Bio-Rad). Data for total and active MMP-2 and MMP-9 in the culture media were normalized against the total protein content of the culture supernatants. The detection limits of the assay are 0.19–12 ng/mL for MMP-2 and 0.125–16 ng/mL for MMP-9. All experiments were performed in duplicate.

### Statistical Analysis

The STATA program version 12 (StataCorp, College Station, Texas, USA) was used for statistical analysis. Comparisons between different groups were made using one-way analysis of variance (ANOVA) following Scheffé’s method, the Mann-Whitney *U* test, or the Wilcoxon matched pairs signed-ranks test. Statistical significance was defined as P<0.05.

## Results

### Effects of ß-catenin siRNA on Cyclin D1, Survivin, and c-Myc Expression and Cell Proliferation

ß-catenin siRNA lowered ß-catenin mRNA and protein expression by approximately 90–95% and 60%, respectively (Figure S2). Expression of Cyclin D1 and Survivin mRNA was significantly decreased, whereas c-Myc and Hyaluronidase-2 (non-Tcf/ß-catenin target gene, negative control) expression was not altered by ß-catenin siRNA (Figure S2). ß-catenin siRNA significantly decreased cell proliferation of endometrial epithelial and stromal cells by approximately 30% and 25%, respectively, whereas no significant decrease was observed in endometriotic epithelial and stromal cells (Figure S2).

### Effects on Cell Proliferation: PKF 115–584 versus CGP049090

The inhibitory effects of PKF 115–584 (6.25 µM) were significantly higher than those with CGP049090 (6.25 µM) in both epithelial and stromal cells prepared from the endometrium at any time of the menstrual cycle (Table S2). However, no significant difference in inhibitory effects was observed for PKF 115–584 versus CGP049090 on endometriotic epithelial and stromal cells (Table S3). Due to the limited number of available cells, only the effects of PKF 115–584 on cell migration and invasion as well as Tcf/ß-catenin target genes were investigated in the present study.

### Endometrium of Patients with and without Endometriosis

#### Effects of PKF 115–584 on cell proliferation

**Figure 1:**
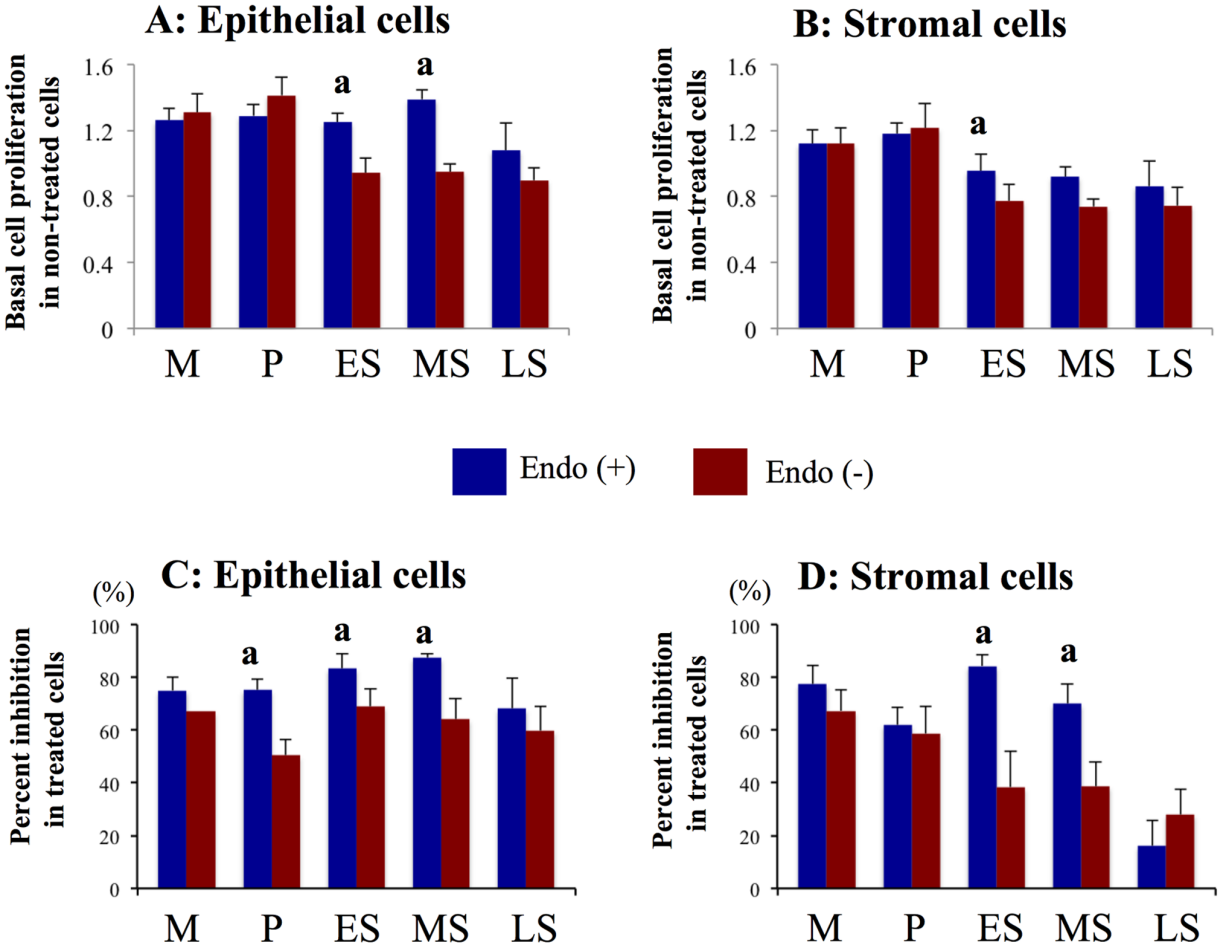
Effects of PKF 115–584 on cell proliferation. A, B: Basal cell proliferation in non-treated endometrial epithelial (A) and stromal (B) cells of patients with and without endometriosis. C, D: Percent inhibition of cell proliferation in endometrial epithelial (C) and stromal (D) cells of patients with and without endometriosis treated with PKF 115–584. Results are presented as the mean+SEM. Basal cell proliferation is presented as OD. Percent inhibition of cell proliferation is calculated as percent of vehicle control. M: menstrual phase, P: proliferative phase, ES: early secretory phase, MS: mid- secretory phase, LS: late secretory phase. Endo (+): Endometrium of patients with endometriosis (M: n = 6, P: n = 20, ES: n = 7, MS: n = 15, LS: n = 6). Endo (–): endometrium of patients without endometriosis (M: n = 4, P: n = 11, ES: n = 8, MS: n = 8, LS: n = 4). a: p<.05 versus patients without endometriosis.

In patients without endometriosis, basal cell proliferation of endometrial cells prepared from the proliferative endometrium was significantly higher than that of epithelial cells from endometrium in other phases, and higher than that of stromal cells from mid- and late-secretory endometrium (Figure 1). In contrast, no significant difference in basal cell proliferation was observed between epithelial or stromal cells prepared from endometrium at different times in the cycle in patients with endometriosis (Figure 1). Basal cell proliferation of epithelial cells prepared from the early- and mid-secretory endometrium and that of stromal cells from the mid-secretory phase was significantly higher in endometrium of patients with endometriosis compared with that of patients without endometriosis (Figure 1).

Inhibition of epithelial cell proliferation by treatment with 6.25 µM PKF 115–584 for 48 h was significantly greater in endometriosis patients compared with that of patients without endometriosis, whereas no significant difference was observed for inhibition of stromal cell proliferation prepared from proliferative endometrium (Figure 1). In addition, inhibition of cell proliferation by treatment with PKF 115–584 in epithelial and stromal cells prepared from the early- and mid-secretory endometrium was significantly greater in endometriosis patients compared with that of patients without endometriosis (Figure 1). However, no significant difference in inhibition of cell proliferation by treatment with PKF 115–584 in epithelial and stromal cells prepared from the menstrual phase was observed between patients with and without endometriosis (Figure 1).

#### Effects of PKF 115–584 on cell migration

**Figure 2:**
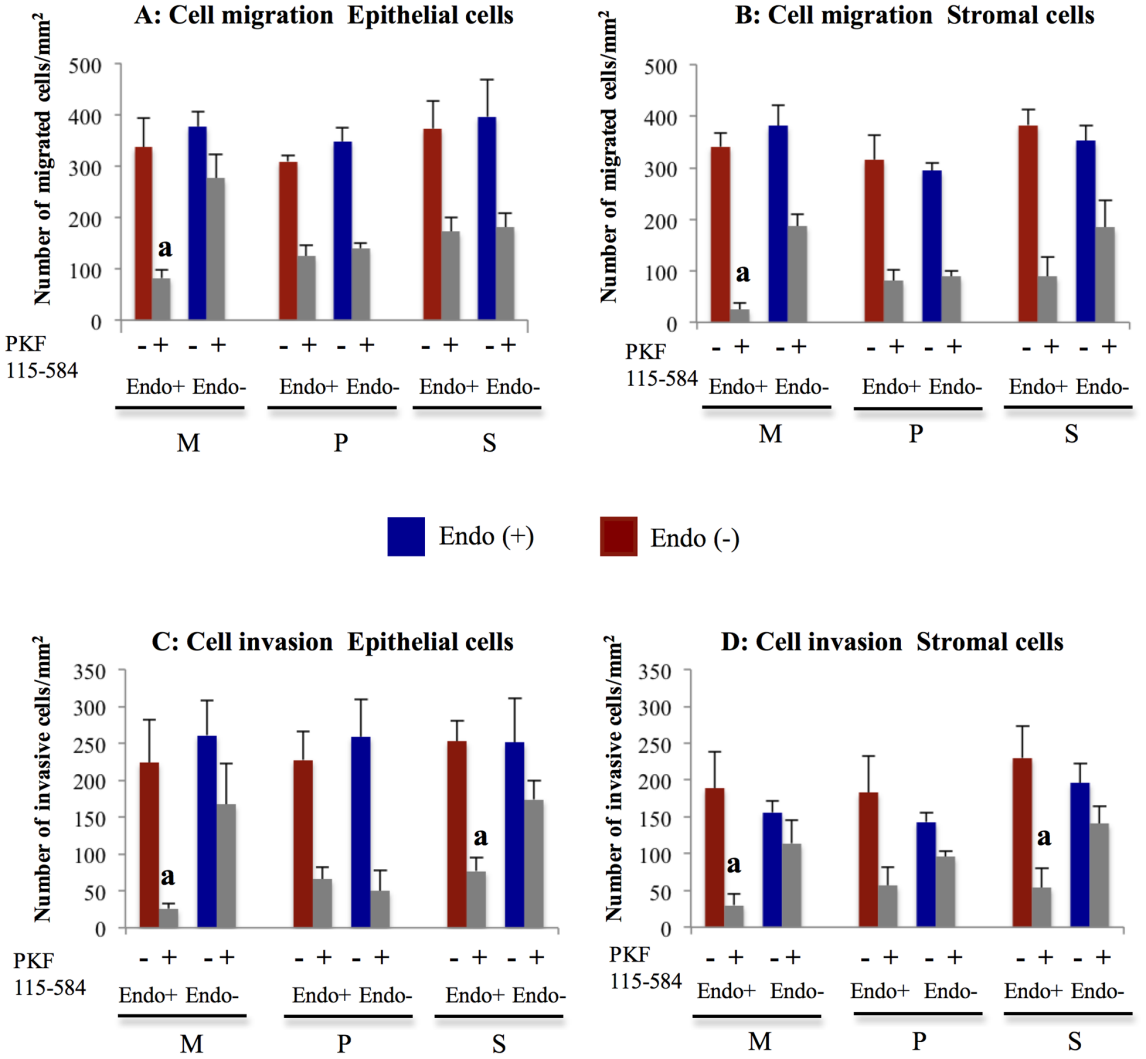
Effects of PKF 115–584 on cell migration and invasion. **A, B**: Number of migrated cells/mm^2^ in non-treated and PKF 115–584–treated endometrial epithelial (A) and stromal (B) cells of patients with and without endometriosis. **C, D**: Number of invasive cells/mm^2^ in non-treated and PKF 115–584–treated endometrial epithelial (C) and stromal (D) cells of patients with and without endometriosis. Results are presented as the mean+SEM. M: menstrual phase, P: proliferative phase, S: secretory phase. Endo (+): Endometrium of patients with endometriosis (M: n = 4, P: n = 8, S: n = 8). Endo (–): endometrium of patients without endometriosis (M: n = 4, P: n = 5, S: n = 5). a: p<.05 versus PKF 115–584–treated endometrial epithelial or stromal cells of patients without endometriosis.

**Figure 3:**
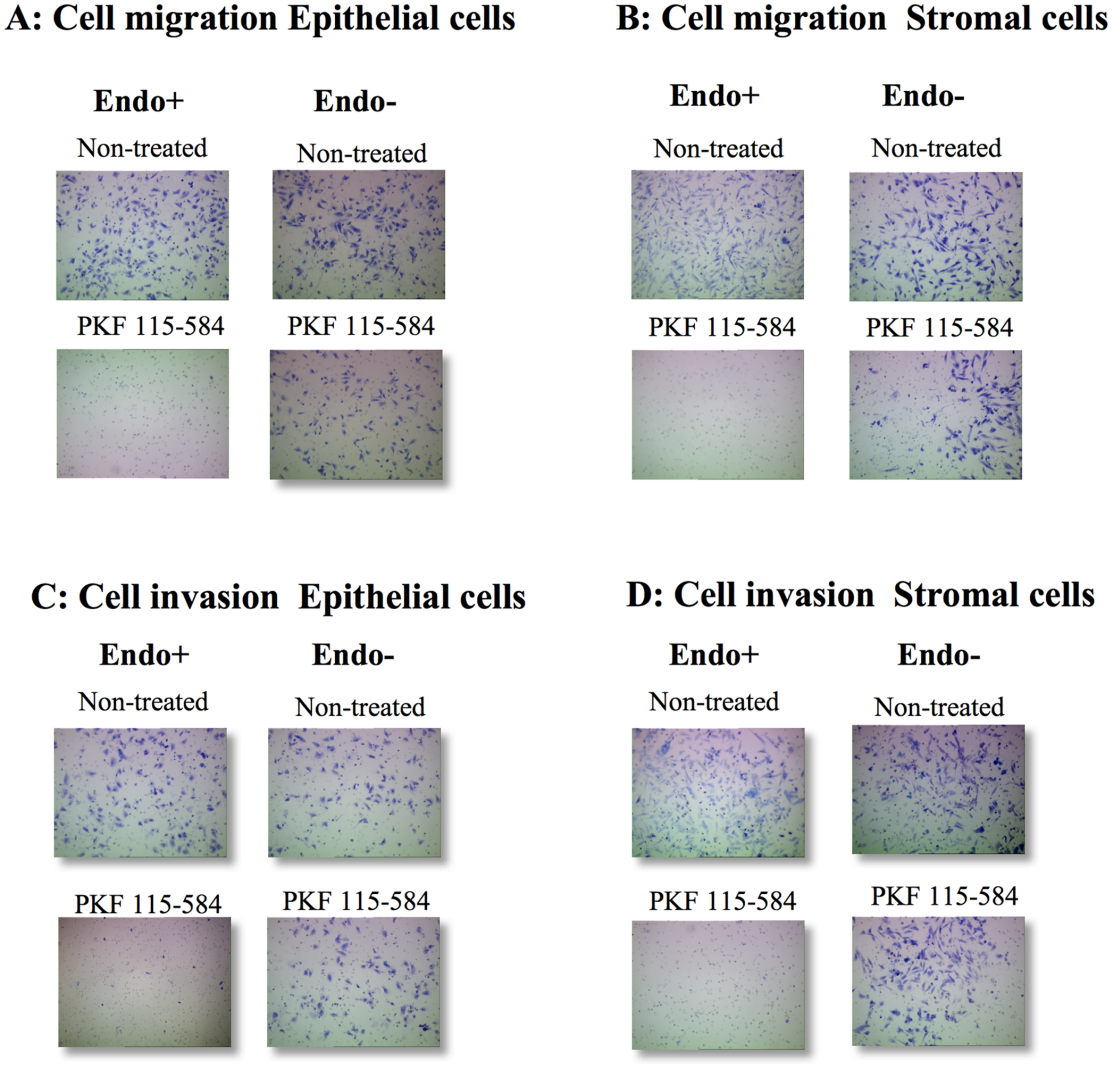
Representative photomicrographs of cell migration and invasion. **A, B**: Representative photomicrographs of migration of non-treated and PKF 115–584–treated menstrual endometrial epithelial (A) and stromal (B) cells of patients with and without endometriosis (magnification x100). **C, D**: Representative photomicrographs of invasion of non-treated and PKF 115–584–treated menstrual endometrial epithelial (C) and stromal (D) cells of patients with and without endometriosis (magnification x100). Endo (+): Endometrium of patients with endometriosis prepared from the menstrual phase. Endo (–): endometrium of patients without endometriosis prepared from the menstrual phase.

In non-treated cells, no significant difference in the number of migrated epithelial and stromal cells prepared from endometrial tissues at different times in the cycle was observed between patients with and without endometriosis (Figures 2 and 3). No significant difference in inhibition of cell migration by treatment with 6.25 µM PKF 115–584 for 24 h in epithelial and stromal cells prepared from either the proliferative or secretory phases was observed between patients with and without endometriosis (Figures 2 and 3). However, in the menstrual phase, inhibition of migration by treatment with PKF 115–584 in epithelial and stromal cells was significantly greater in endometriosis patients than in patients without endometriosis (Figures 2 and 3).

#### Effects of PKF 115–584 on cell invasion

In non-treated cells, no significant difference was noted in the number of invasive epithelial and stromal cells prepared from endometrial tissues at different times in the cycle between patients with and without endometriosis (Figures 2 and 3). Furthermore, no significant difference in inhibition of cell invasion by treatment with 6.25 µM PKF 115–584 for 24 h in epithelial and stromal cells prepared from the proliferative endometrium was observed between patients with and without endometriosis (Figures 2 and 3). However, inhibition of cell invasion by treatment with PKF 115–584 in epithelial and stromal cells prepared from the secretory and menstrual phases was significantly greater in patients with endometriosis than in patients without endometriosis (Figures 2 and 3).

#### Effects of PKF 115–584 on Tcf/ß-catenin target genes

**Figure 4:**
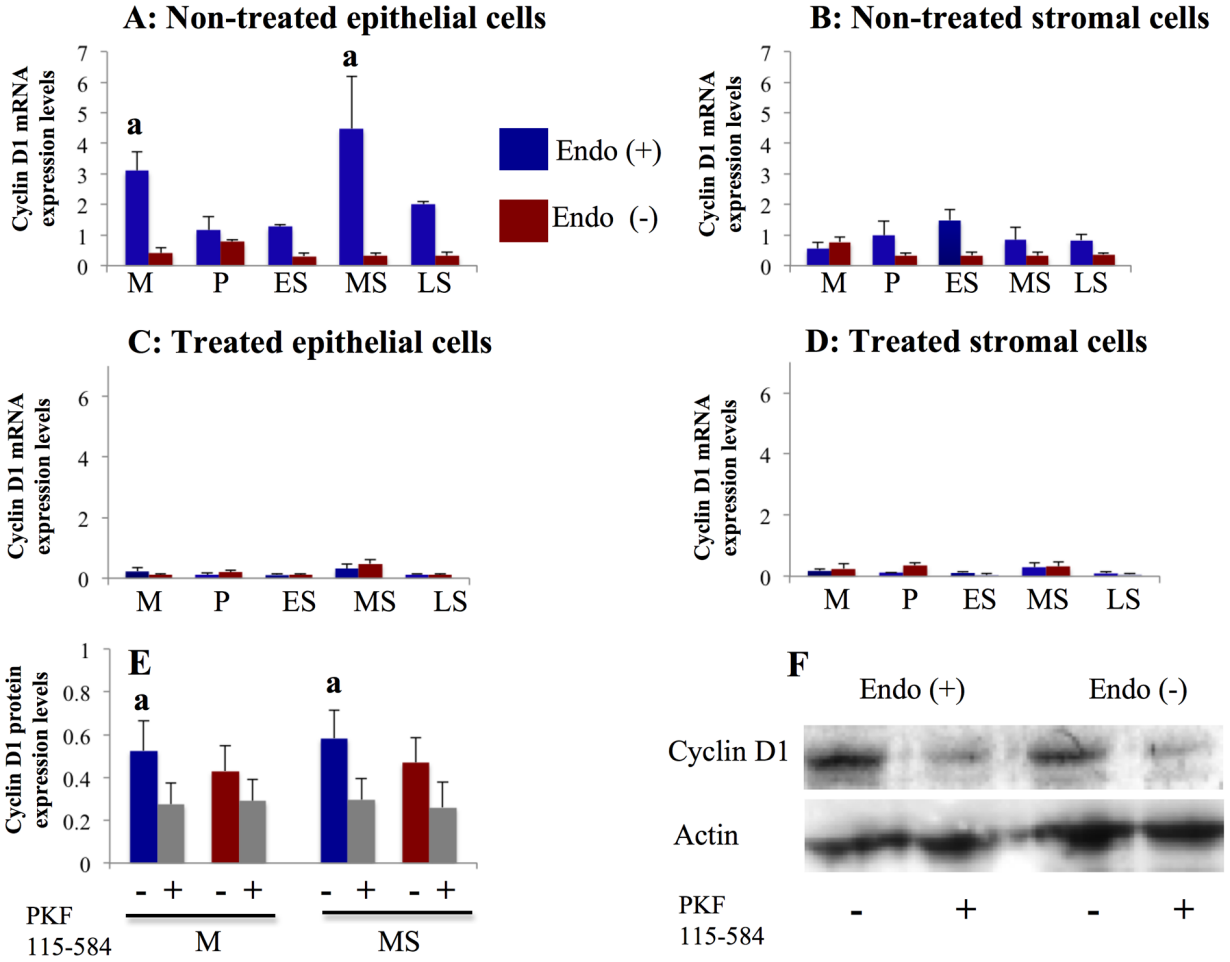
Effects of PKF 115–584 on Cyclin D1 expression. **A, B**: Cyclin D1 mRNA expression in non-treated endometrial epithelial (A) and stromal (B) cells of patients with and without endometriosis. Endo (+) (M: n = 6, P: n = 20, ES: n = 7, MS: n = 15, LS: n = 6). Endo (–) (M: n = 4, P: n = 11; ES: n = 8, MS: n = 8; LS: n = 4). **C, D**: Cyclin D1 mRNA expression in PKF 115–584–treated endometrial epithelial (C) and stromal (D) cells of patients with and without endometriosis. Endo (+):(M: n = 6, P: n = 20, ES: n = 7, MS: n = 15, LS: n = 6). Endo (–):(M: n = 4, P: n = 11, ES: n = 8, MS: n = 8, LS: n = 4). **E**: Cyclin D1 protein expression in non-treated and PKF 115–584–treated endometrial epithelial cells from the mid-secretory and menstrual phases. Endo (+):(M: n = 4, MS: n = 5). Endo (–):(M: n = 4, MS: n = 5). **F**: Representative photomicrographs of western blot analysis in non-treated and PKF 115–584–treated endometrial epithelial from the mid-secretory phase. Numerical values are presented as the mean+SEM. Expression levels of Cyclin D1 mRNA are given relative to the expression levels of the reference gene, GAPDH. Relative density is density of Cyclin D1 relative to that of Actin. M: menstrual phase, P: proliferative phase, ES: early secretory phase, MS: mid- secretory phase, LS: late secretory phase. a: p<.05 versus patients without endometriosis.

**Figure 5:**
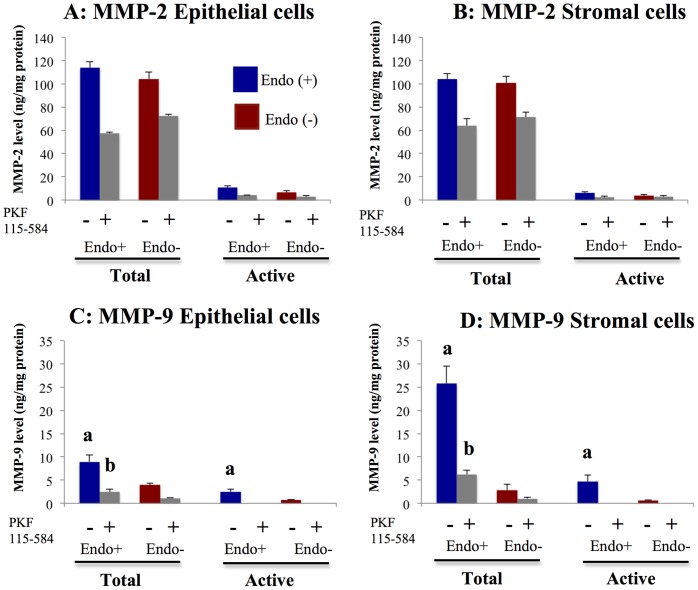
Effects of PKF 115–584 on total and active forms of MMP-2 and MMP-9. **A, B**: Total and active forms of MMP-2 in non-treated and PKF 115–584–treated endometrial epithelial (A) and stromal (B) cells of patients with and without endometriosis. **C, D**: Total and active forms of MMP-9 in non-treated and PKF 115–584–treated endometrial epithelial (C) and stromal (D) cells of patients with and without endometriosis. Endo (+): Endometrium of patients with endometriosis (epithelial cells: n = 4, stromal cells: n = 4). Endo (–): endometrium of patients without endometriosis (epithelial cells: n = 3, stromal cells: n = 3). Values are normalized to the total protein content of the culture supernatants. Results are presented as the mean+SEM. a: p<.05 versus non-treated endometrial epithelial or stromal cells of patients without endometriosis. b: p<.05 versus PKF 115–584–treated endometrial epithelial or stromal cells of patients without endometriosis.MMP-9

Expression of Cyclin D1 (Figure 4), Survivin (Table S4), MMP-2 (Figure 5, Table S5), and MMP-9 (Figure 5, Table S6) mRNA was significantly decreased following treatment with PKF 115–584, whereas expression of c-Myc (Table S7) and Hyaluronidase-2 (non–Tcf/ß-catenin target gene, Table S8) mRNA was not altered.

#### Cyclin D1

Cyclin D1 mRNA expression levels in non-treated epithelial cells prepared from the mid-secretory and menstrual phases were significantly higher in patients with endometriosis than in patients without endometriosis (Figure 4). However, no significant difference in Cyclin D1 mRNA expression in non-treated stromal cells prepared from endometrial tissues at different times in the cycle was observed between patients with and without endometriosis (Figure 4). In addition, no significant difference in Cyclin D1 mRNA expression in epithelial and stromal cells prepared from endometrial tissue at different times in the cycle treated with PKF 115–584 for 24 h was noted between patients with and without endometriosis (Figure 4). Western blot analysis revealed that Cyclin D1 protein expression levels in non-treated epithelial cells prepared from the mid-secretory and menstrual phases were significantly higher in patients with endometriosis than in patients without endometriosis (Figure 4). No significant difference in Cyclin D1 protein expression was observed in PKF 115–584–treated epithelial cells prepared from the mid-secretory and menstrual phases between patients with and without endometriosis (Figure 4).

#### Survivin

No significant differences in Survivin mRNA expression in either non-treated epithelial or stromal cells were observed between patients with and without endometriosis. Furthermore, no significant differences in Survivin mRNA expression in either non-treated epithelial or stromal cells prepared from different times in the cycle were observed in patients with and without endometriosis. Thus, we analyzed the effects of PKF 115–584 on Survivin mRNA expression irrespective of menstrual phase. No significant differences in Survivin mRNA expression in epithelial and stromal cells treated with PKF 115–584 were observed between patients with and without endometriosis (Table S4).

#### MMP-2

MMP-2 mRNA expression was significantly higher in epithelial cells prepared from the menstrual endometrium than from the other phases in the cycle in patients with endometriosis (Table S5). In contrast, no significant differences in MMP-2 mRNA expression in epithelial cells prepared from different times in the cycle were observed in patients without endometriosis (Table S5). Furthermore, no significant difference in MMP-2 mRNA expression in stromal cells prepared from different times in the cycle was observed between patients with and without endometriosis (Table S5). MMP-2 mRNA expression levels in epithelial cells prepared from the menstrual phase were significantly higher in patients with endometriosis than in patients without endometriosis, whereas no significant difference was observed for the other phases in the cycle (Table S5). In addition, no significant difference in MMP-2 mRNA expression in epithelial and stromal cells prepared from different times in the cycle treated with PKF 115–584 was noted between patients with and without endometriosis (Table S5).

No significant difference in total and active forms of MMP-2 was observed in either non-treated or treated epithelial and stromal cells prepared from the menstrual endometrium between patients with and without endometriosis (Figure 5).

MMP-9 mRNA expression in epithelial cells prepared from menstrual endometrium was significantly higher than that from endometrium in other phases in patients with endometriosis (Table S6). In contrast, no significant differences in MMP-9 mRNA expression in epithelial cells prepared from different times in the cycle were observed in patients without endometriosis (Table S6). Moreover, no significant difference was observed in stromal cells prepared from different times in the cycle between patients with and without endometriosis (Table S6). No significant difference in MMP-9 mRNA in either treated or non-treated epithelial and stromal cells prepared from different times in the cycle was observed between patients with and without endometriosis (Table S6).

Total and active forms of MMP-9 were significantly higher in epithelial and stromal cells prepared from menstrual endometrium in patients with endometriosis compared to patients without endometriosis (Figure 5). In epithelial and stromal cells prepared from the menstrual endometrium treated with PKF 115–584, total MMP-9 was significantly higher in patients with endometriosis than in patients without endometriosis (Figure 5). No significant difference in the amount of active MMP-9 in epithelial and stromal cells treated with PKF 115–584 was observed between patients with and without endometriosis (Figure 5).

### Endometriotic Tissue Versus Matched Eutopic Endometrium of the Same Patients

#### Effects of PKF 115–584 on cell proliferation

**Figure 6:**
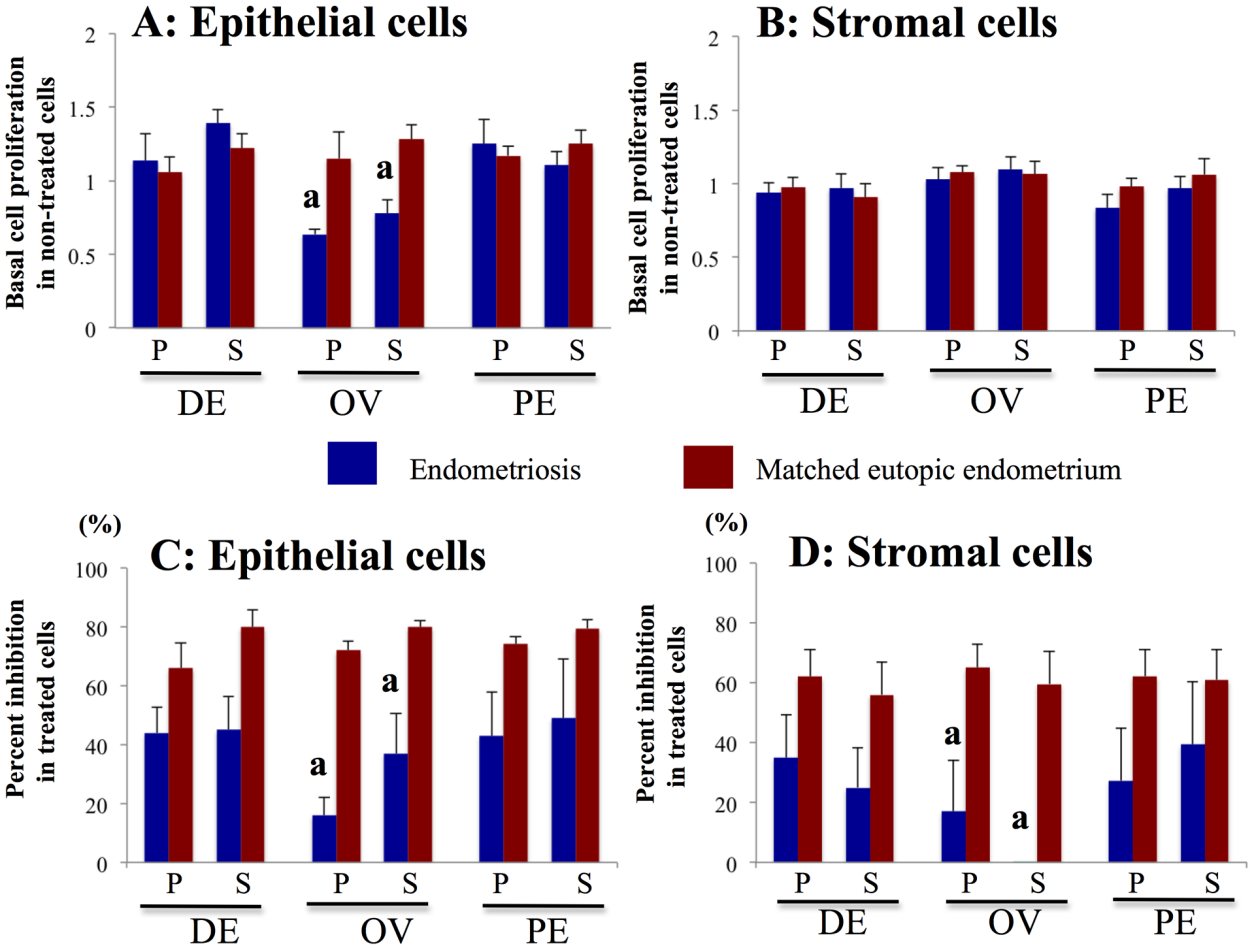
Effects of PKF 115–584 on cell proliferation. **A, B**: Basal cell proliferation in non-treated epithelial (A) and stromal (B) cells of endometriotic tissue and matched eutopic endometrium of the same patients. **C, D**: Percent inhibition of cell proliferation in epithelial (C) and stromal (D) cells of endometriotic tissue and matched eutopic endometrium of the same patients treated with PKF 115–584. Results are presented as the mean+SEM. Basal cell proliferation is presented as OD. Percent inhibition of cell proliferation is calculated as percent of vehicle control. P: proliferative phase, S: secretory phase. DE: deep infiltrating endometriosis (epithelial cells: P: n = 7, S: n = 8; stromal cells: P: n = 7, S:n = 9 ). OE: ovarian endometriosis (epithelial cells: P: n = 7 S: n = 6; stromal cells: P: n = 7, S: n = 8 ). SE: superficial peritoneal endometriosis (epithelial cells: P: n = 6, S: n = 6; stromal cells: P: n = 6, S: n = 7 ). A: p<.05 versus matched eutopic endometrium of the same patients.

No significant difference in basal cell proliferation was observed between endometriotic epithelial cells and matched eutopic endometrial epithelial cells of the same patients prepared from deep infiltrating endometriotic tissue or superficial peritoneal endometriotic tissue (Figure 6). However, basal epithelial cell proliferation of ovarian endometriotic tissue was significantly lower than that of matched eutopic endometrium of the same patients (Figure 6). No significant difference in basal stromal cell proliferation was observed between endometriotic tissue and matched eutopic endometrium of the same patients (Figure 6). Inhibition of cell proliferation by treatment with PKF 115–584 was significantly lower in both epithelial and stromal cells of ovarian endometriotic tissue than that of matched eutopic endometrium from the same patients (Figure 6). In contrast, no significant difference in inhibition of cell proliferation by treatment with PKF 115–584 in either epithelial or stromal cells was noted between the other types of endometriotic tissue (deep infiltrating endometriosis or superficial peritoneal endometriosis) and matched eutopic endometrium of the same patients (Figure 6).

#### Effects of PKF 115–584 on cell migration

**Figure 7:**
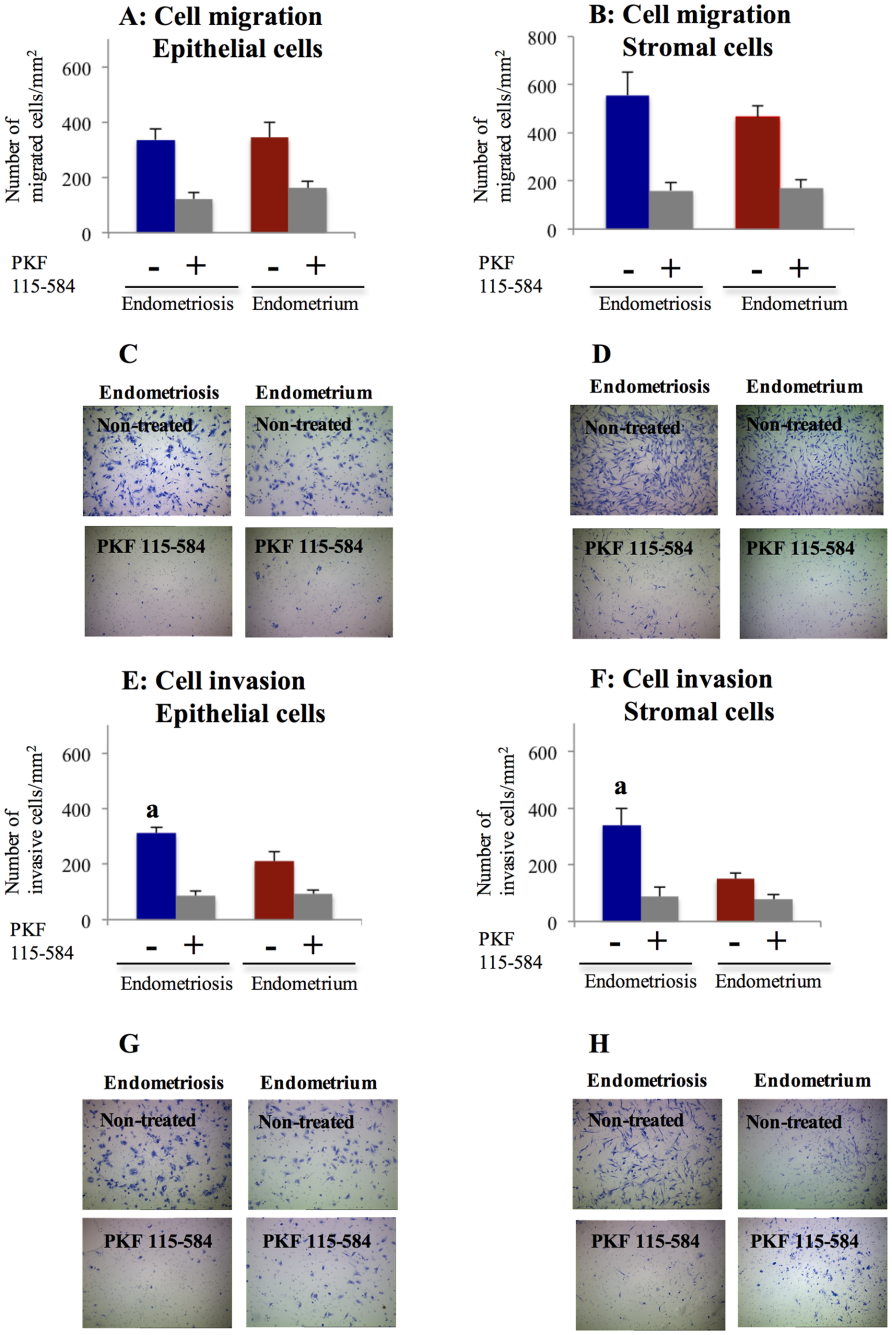
Effects of PKF 115–584 on cell migration and invasion. **A, B**: Number of migrated cells/mm^2^ in non-treated and PKF 115–584–treated epithelial (A) and stromal (B) cells of endometriotic tissue and matched eutopic endometrium of the same patient. **C, D**: Representative photomicrographs of migration of non-treated and PKF 115–584–treated epithelial (C) and stromal (D) cells of endometriotic tissue and matched eutopic endometrium of a same patient (magnification x100). **E, F**: Number of invasive cells/mm^2^ in non-treated and PKF 115–584–treated epithelial (E) and stromal (F) cells of endometriotic tissue and matched eutopic endometrium of the same patients. **G, H**: Representative photomicrographs of invasion of non-treated and PKF 115–584–treated epithelial (G) and stromal (H) cells of endometriotic tissue and matched eutopic endometrium of a same patient (magnification x100). Results are presented as the mean+SEM. Epithelial cells of endometriotic tissue and matched eutopic endometrium of the same patients (n = 16). Stromal cells of endometriotic tissue and matched eutopic endometrium (n = 16). a: p<.05 versus matched eutopic endometrium of the same patients.

In either non-treated or treated cells, no significant difference in the number of migrated epithelial and stromal cells was observed between endometriotic tissue and matched eutopic endometrium of the same patients (Figure 7). In addition, no significant difference in percent inhibition of cell migration by treatment with PKF 115–584 in either epithelial or stromal cells was noted between endometriotic tissue and eutopic endometrium of the same patients (Figure 7).

#### Effects of PKF 115–584 on cell invasion

In non-treated cells, the number of invasive cells was significantly higher in epithelial and stromal cells of endometriotic tissue compared with those of matched eutopic endometrium of the same patients (Figure 7). The percent inhibition was significantly higher in endometriotic epithelial and stromal cells compared with those of eutopic endometrium of the same patients (Figure 7). In PKF 115–584–treated cells, no significant difference in the number of invasive cells was observed between endometriotic tissue and matched eutopic endometrium of the same patients (Figure 7).

#### Effects of PKF 115–584 on Tcf/ß-catenin target genes

**Figure 8:**
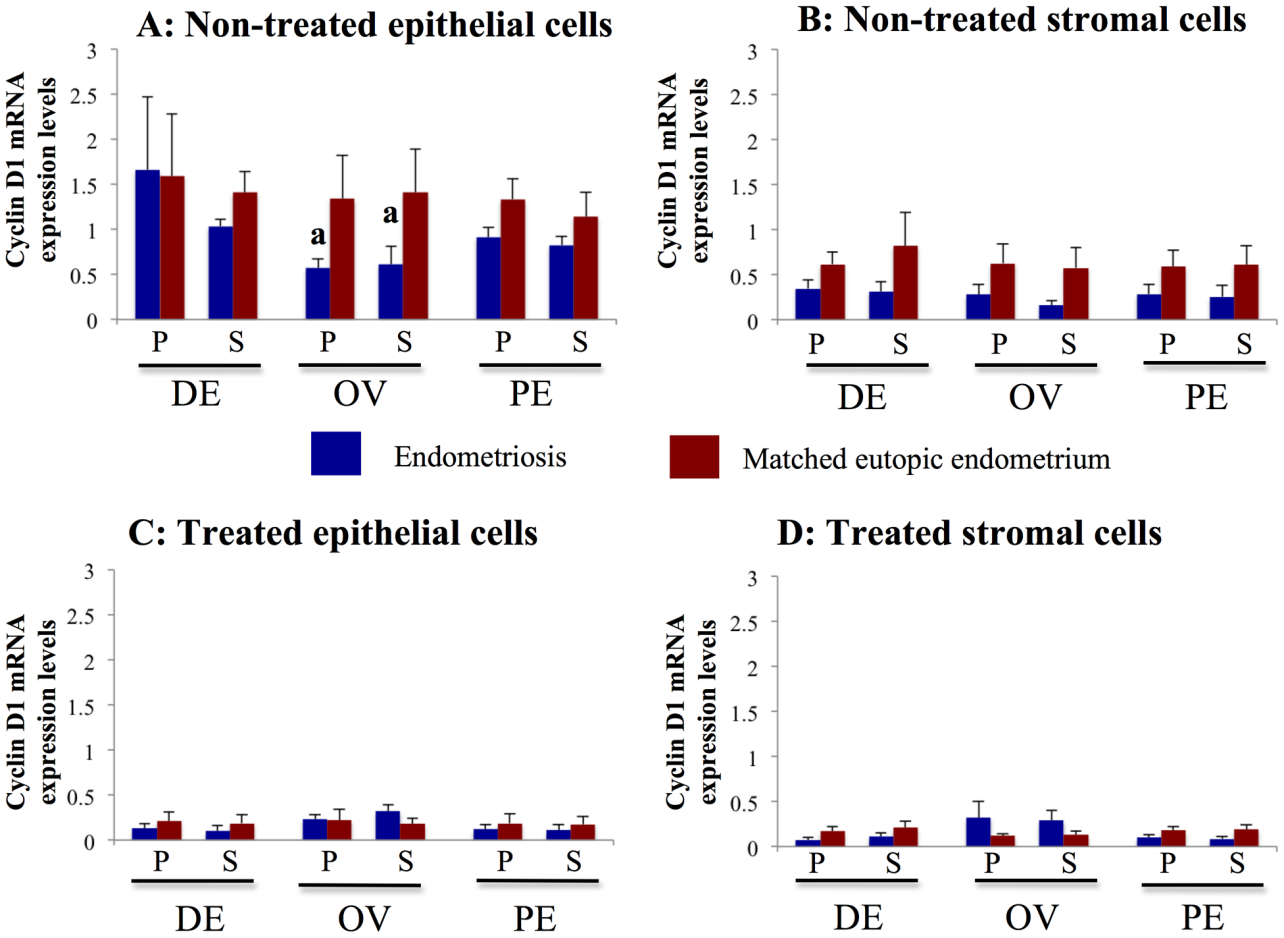
Effects of PKF 115–584 on Cyclin D1 expression. **A, B**: Cyclin D1 mRNA expression in non-treated epithelial (A) and stromal (B) cells of endometriotic tissue and matched eutopic endometrium of the same patients. **C, D**: Cyclin D1 mRNA expression in PKF 115–584–treated epithelial (C) and stromal (D) cells of endometriotic tissue and matched eutopic endometrium of the same patients. Numerical values are presented as the mean+SEM. Expression levels of Cyclin D1 mRNA are given relative to the expression levels of the reference gene, GAPDH. P: proliferative phase, S: secretory phase. DE: deep infiltrating endometriosis (epithelial cells: P: n = 6, S: n = 6, stromal cells: P: n = 6, S: n = 6). OE: ovarian endometriosis (epithelial cells: P: n = 6, S: n = 6; stromal cells: P: n = 6, S: n = 6). SE: superficial peritoneal endometriosis (epithelial cells: P: n = 4, S: n = 4; stromal cells: P: n = 4, S: n = 4). a: p<.05 versus matched eutopic endometrium of the same patients.

**Figure 9:**
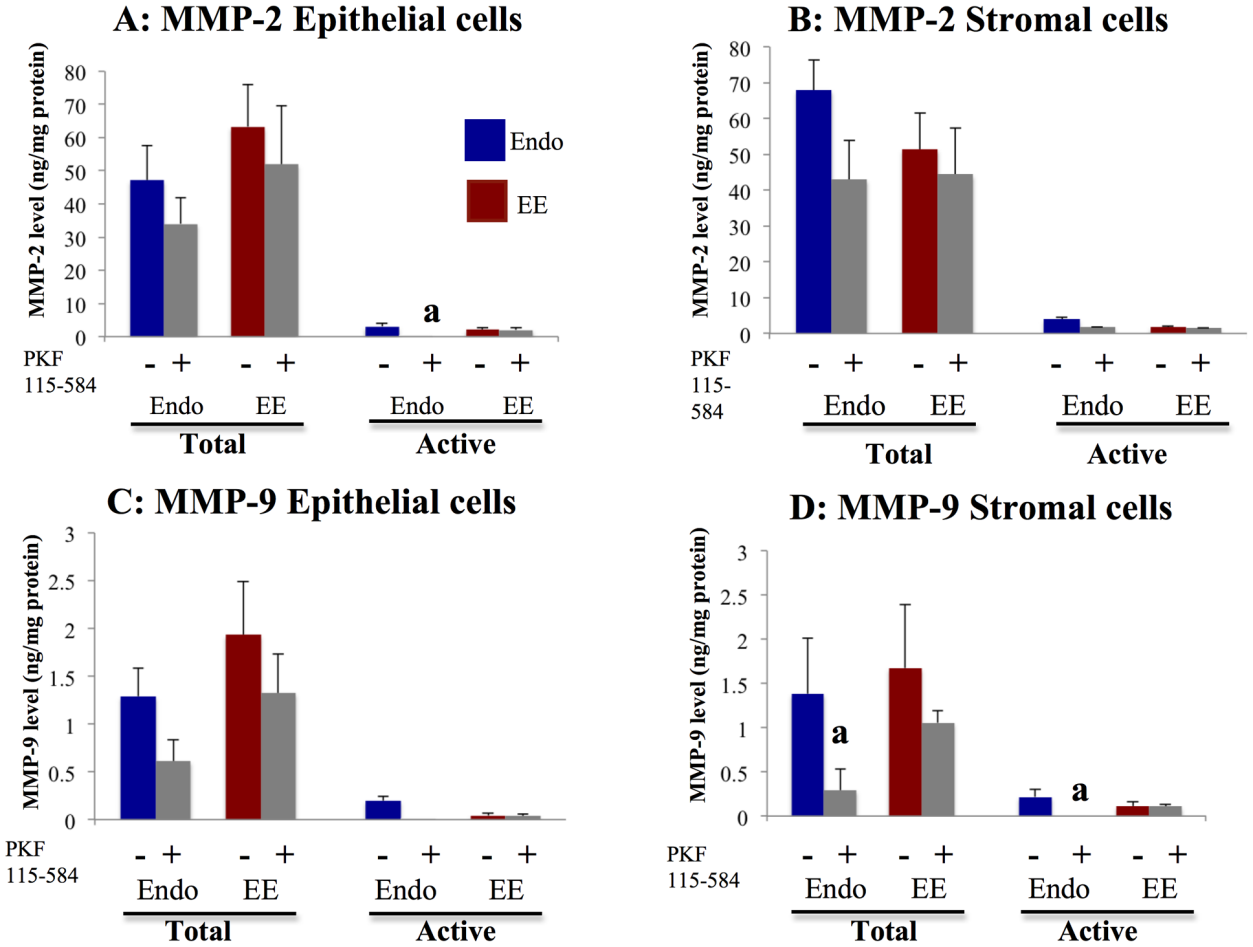
Effects of PKF 115–584 on total and active forms of MMP-2 and MMP-9. **A, B**: Total and active forms of MMP-2 in non-treated and PKF 115–584–treated epithelial (A) and stromal (B) cells of endometriotic tissue and matched eutopic endometrium of patients with endometriosis. **C, D**: Total and active forms of MMP-9 in non-treated and PKF 115–584–treated epithelial (C) and stromal (D) cells of endometriosis and matched eutopic endometrium of patients with endometriosis. Values are normalized to the total protein content of the culture supernatants. Results are presented as the mean +SEM. Endo: endometriosis, EE: matched eutopic endometrium. Endo: (epithelial cells: n = 6, stromal cells: n = 6). EE: (epithelial cells: n = 6, stromal cells: n = 6). a: p<.05 versus PKF 115–584–treated endometrial epithelial or stromal cells.

Expression of Cyclin D1 (Figure 8), Survivin (Table S9), MMP-2 (Figure 9, Table S10), and MMP-9 (Figure 9, Table S11) mRNA was significantly decreased following treatment with PKF 115–584, whereas expression of c-Myc mRNA (Table S12) and Hyaluronidase-2 (non–Tcf/ß-catenin target gene, Table S8) mRNA was not altered.

#### Cyclin D1

Expression levels of Cyclin D1 mRNA in non-treated epithelial cells were significantly lower in ovarian endometriotic tissue than those of matched eutopic endometrium (Figure 8). In contrast, no significant difference in Cyclin D1 expression in non-treated epithelial cells was observed between the other types of endometriotic tissue (deep endometriosis and superficial peritoneal endometriosis) and matched eutopic endometrium of the same patients (Figure 8). In addition, no significant difference in Cyclin D1 expression of non-treated stromal cells was noted between endometriotic tissue and matched eutopic endometrium of the same patients (Figure 8). In cells treated with PKF 115–584, no significant difference in Cyclin D1 mRNA expression in either epithelial or stromal cells was observed between endometriotic tissue and matched eutopic endometrium of the same patients (Figure 8).

#### Survivin

No significant cyclical differences in Survivin mRNA expression in either epithelial or stromal cells were observed in endometriotic tissues. Thus, we analyzed the effects of PKF 115–584 on Survivin mRNA expression irrespective of menstrual phase. No significant difference in Survivin mRNA expression in either non-treated or treated epithelial and stromal cells was observed between endometriotic tissue and matched eutopic endometrium of the same patients (Table S9).

#### MMP-2

No significant difference in MMP-2 mRNA expression in either non-treated or treated epithelial and stromal cells was observed between endometriotic tissue and matched eutopic endometrium of the same patients (Table S10). No significant difference in total and active forms of MMP-2 in epithelial and stromal cells was observed between endometriotic tissue and matched eutopic endometrium of the same patients (Figure 9). Levels of active MMP-2 were significantly lower after treatment with PKF 115–584 in epithelial cells of endometriotic tissue compared with those of matched eutopic endometrium (Figure 9). However, no significant difference in levels of total and active MMP-2 in stromal cells after treatment was noted between endometriotic tissue and matched eutopic endometrium.

#### MMP-9

No significant difference in MMP-9 mRNA expression in either non-treated or treated epithelial and stromal cells was observed between endometriotic tissue and matched eutopic endometrium of the same patients (Table S11). No significant difference in total or active MMP-9 in non-treated epithelial and stromal cells was noted between endometriotic tissue and matched eutopic endometrium of the same patients (Figure 9). In addition, no significant difference in total or active MMP-9 in epithelial cells treated with PKF 115–584 was observed between endometriotic tissue and matched eutopic endometrium (Figure 9). However, levels of total and active MMP-9 were significantly lower in endometriotic stromal cells treated with PKF 115–584 compared to those of matched eutopic endometrium of the same patients (Figure 9).

## Discussion

The results of the present study demonstrated that the inhibitory effects of cell migration and invasion of menstrual endometrial epithelial and stromal cells of endometriosis patients were much higher than those of patients without endometriosis. Since cell proliferation, migration, and invasion of endometrial and endometriotic cells were inhibited by PKF 115–584, it is possible that the inhibition of invasion and migration was the result of inhibition of proliferation. However, in the present study, no significant differences in the inhibitory effects of cell proliferation of menstrual epithelial and stromal cells were observed between patients with and without endometriosis. The present study also revealed that menstrual epithelial and stromal cells of patients with endometriosis possessed significantly higher total and active forms of MMP-9 compared to those of patients without endometriosis. Treatment with PKF 115–584 decreased the amount of total MMP-9 approximately 75% in epithelial cells and 85% in stromal cells in patients with endometriosis. Furthermore, treatment with PKF 115–584 decreased the amount of active MMP-9 to undetectable levels in both epithelial and stromal cells of patients with endometriosis. MMP-9 activity is known to be involved in cell invasion [21]–[24]. In addition, recent studies clearly demonstrated that a latent form of MMP-9 may play an important role in cell migration [25], [26]. These findings suggested that much higher levels of total and active MMP-9 in endometrial epithelial and stromal cells of patients with endometriosis during the menstrual phase might be involved in the pathophysiology of endometriosis. The MMP-2 protein is found in endometrial tissue in both latent and active forms throughout the cycle [27]. However, the active form is more abundant at menstruation. In addition, active MMP-9 is exclusively seen at menstruation [27]. Therefore, in the present study, we focused on menstrual endometrium to investigate total and active forms of MMP-2 and MMP-9. The present findings are consistent with those of a previous study that demonstrated that MMP-9 secretion, as assessed by zymography and enzyme-linked immunosorbent assay (ELISA), was increased in women with endometriosis compared to healthy women, while no statistically significant difference in MMP-2 secretion was observed [28]. According to the implantation theory, two processes appear to be critical for the establishment of endometriosis: migration and invasion [1], [29]. The present findings suggested that the Wnt/ß-catenin signaling pathway may represent a novel therapeutic target for prevention of endometriosis.

Previous studies have suggested that progesterone resistance might result in failure to inhibit activation of Wnt/ß-catenin signaling, resulting in the persistence of the proliferative phenotype in the endometrium of infertile patients with endometriosis during the window of implantation [2], [30]–[33]. In the present study, basal cell proliferation of epithelial cells prepared from the early-, mid-, and late-secretory endometrium was observed to be significantly lower than that from the proliferative endometrium in patients without endometriosis. However, in patients with endometriosis, no significant differences in basal cell proliferation of epithelial cells prepared from endometrium were observed at different times in the cycle. Basal cell proliferation of endometrial epithelial and stromal cells prepared from the mid-secretory endometrium was significantly higher in patients with endometriosis compared to patients without endometriosis. In addition, the present results demonstrated significantly higher expression of Cyclin D1, a Tcf/ß-catenin target gene, in endometrial epithelial cells of patients with endometriosis compared to patients without endometriosis in the mid-secretory phase. Although no significant difference in Cyclin D1 expression in endometrial stromal cells was detected between patients with and without endometriosis, expression levels tended to be higher in patients with endometriosis compared to patients without endometriosis during the secretory phase, which is consistent with the results of a previous study [33]. Treatment with PKF 115–584 effectively decreased cell proliferation and Cyclin D1 expression in endometrial epithelial and stromal cells of patients with endometriosis. These findings may further support previous findings, including those from our laboratory, of aberrant activation of Wnt/ß-catenin signaling, resulting in the persistence of the proliferative phenotype in the endometrium of infertile patients with endometriosis during the window of implantation [2], [31]–[33].

In the present study, the inhibitory effect of treatment on cell proliferation in ovarian endometriotic tissue was significantly lower than that of matched eutopic endometrium of the same patients. In the present study, basal cell proliferation of epithelial cells and Cyclin D1 mRNA expression of ovarian endometriotic tissue were significantly lower than those of matched eutopic endometrium of the same patients. Borghese *et al*. demonstrated a systemic down-regulation of genes involved in the cell cycle in ovarian endometriosis, using the NimbleGen platform that contains 47,633 transcripts [34]. Treatment with PKF 115–584 may have had little effect on the inhibition of cell proliferation of ovarian endometriosis because endometrioma may be quiescent, as speculated by Borghese *et al.* [34]. Thus, therapies that target cell proliferation may not be effective in ovarian endometriosis. In contrast, no significant difference in basal cell proliferation or Cyclin D1 mRNA expression was observed in deep infiltrating endometriotic tissue and superficial peritoneal endometriotic tissue compared with those of matched eutopic endometrium of the same patients. Furthermore, no statistically significant difference was observed in percent inhibition of cell proliferation, Cyclin D1 or Survivin mRNA expression in treated epithelial and stromal cells between endometriotic tissue and eutopic endometrium of the same patients. However, the cell proliferation inhibitory effect tended to be lower in endometriotic tissue than in eutopic endometrium of the same patients. These findings suggested that the Wnt/ß-catenin signaling pathway might not be essential for cell proliferation of endometriotic cells. In addition, Cyclin D1 and Survivin might not be essential for the regulation of endometriotic cell proliferation.

In the present study, the numbers of invasive epithelial and stromal cells of endometriotic tissues were significantly higher than those of matched eutopic endometrium of the same patients. These findings are consistent with the results of previous studies that showed that the invasive phenotype in endometriosis shares aspects with tumor metastasis [35], [36]. Clinical observations and in vitro experiments imply that endometriotic cells are invasive and able to metastasize [35], [36]. Treatment with PKF 115–584 significantly decreased the numbers of invasive endometriotic epithelial and stromal cells to levels similar to those of matched eutopic endometrium. In the present study, levels of active MMP-2 in endometriotic epithelial cells and total and active MMP-9 in endometriotic stromal cells were significantly decreased compared to those of matched eutopic endometrium following treatment with PKF 115–584. In addition, levels of active MMP-2 in stromal cells and active MMP-9 in treated epithelial cells tended to be lower in endometriotic tissues compared with those of eutopic endometrium of the same patients. These findings suggest that the numbers of invasive endometriotic epithelial cells and stromal cells were significantly decreased following treatment with PKF 115–584, partly through inhibition of active MMP-2 and MMP-9. A recent study demonstrated that the activated TNF­α–MMP-9 axis processes SRC­1 to its C­terminal isoform to protect the ectopic endometrium from proinflammatory cytokine­mediated cell death, which is then accompanied by epithelial-to-mesenchymal transition (EMT) and enhancement of the invasive capacity of the ectopic endometrium [37]. Furthermore, endometrial expression of TNF-α mRNA was significantly higher during the menstrual phase in women with endometriosis compared to women without endometriosis [38]. We previously demonstrated that EMT might be involved in the pathophysiology of endometriosis [3]. The present results demonstrated that inhibition of Wnt/β-catenin signaling decreased MMP-9 activity in endometriotic tissue and menstrual endometrium. Thus, inhibition of the Wnt/ß-catenin signaling pathway might also inhibit the TNF­α–MMP-9 axis [37]. In the present study, no significant difference in the number of migrated epithelial and stromal cells was observed between endometriotic tissue and matched eutopic endometrium of the same patients before and after treatment. However, approximately 65% and 72% inhibition of migrated cells was observed in epithelial and stromal cells of endometriotic tissues, respectively. These findings suggested that the Wnt/ß-catenin signaling pathway may represent a novel therapeutic target for endometriosis.

### Conclusion

The present study demonstrated that inhibitory effects of cell migration and invasion in endometrial epithelial and stromal cells of patients with endometriosis prepared from the menstrual phase were significantly higher than those of patients without endometriosis. In addition, treatment with a small-molecule antagonist of the Tcf/ß-catenin complex decreased the number of invasive endometriotic epithelial and stromal cells to levels similar to those of matched endometrium. The present findings demonstrated that cellular mechanisms known to be involved in endometriotic lesion development are inhibited by targeting the Wnt/ß-catenin pathway. Further preclinical research is required to investigate whether inhibition of the Wnt/ß-catenin signaling pathway may be effective in the prevention and treatment of endometriosis.

## Supporting Information

Figure S1 **Representative photomicrographs of immunocytochemistry for cytokeratin (A, E), vimentin (B, F), factor VIII (C, G) and CD 45 (D, H) in isolated endometrial epithelial (A–D) and stromal cells (E–H).** Original magnification: ×400. Percentage of cytokeratin, vimentin, factor VIII or CD 45 positive epithelial and stromal cells. ^a^: Although vimentin is a mesenchymal marker, it is also expressed in mesoderm-derived epithelium, such as endometrium (3).Vimentin is also expressed in endometriotic epithelial cells (3, 36).
(TIFF)

Figure S2 **A**: ß-catenin mRNA expression in untransfected (U), control (C) or ß-catenin siRNA-transfected (ß) cells. **B**: Western blot analysis of ß-catenin protein expression in control (C) or ß-catenin siRNA-transfected (ß) endometrial stromal cells (n = 5) and representative photomicrographs of western blot analysis. **C:** Cyclin D1 mRNA expression in untransfected (U), control (C) or ß-catenin siRNA-transfected (ß) cells. **D**: Survivin mRNA expression in untransfected (U), control (C) or ß-catenin siRNA-transfected (ß) cells. **E**: c-Myc mRNA expression in untransfected (U), control (C) or ß-catenin siRNA-transfected (ß) cells. **F**: Hyaluronidase-2 (Hyal-2) mRNA expression in untransfected (U), control (C) or ß-catenin siRNA-transfected (ß) cells. **G:** Cell proliferation in untransfected (U), control (C) or ß-catenin siRNA-transfected (ß) cells. Numerical values are presented as the mean+SEM. Expression levels of ß-catenin, Cyclin D1, Survivin, c-Myc mRNA and Hyaluronidase-2 are given relative to the expression levels of the reference gene, GAPDH. ß-catenin protein expression in ß-catenin siRNA-transfected cells (ß) was normalized to respective controls (C). Cell proliferation in control (C) or ß-catenin siRNA-transfected (ß) cells was normalized to untransfected (U) cells. EEE: endometrial epithelial cells of patients with endometriosis (proliferative phase: n = 10). EES: endometrial stromal cells of patients with endometriosis (proliferative phase: n = 10). ENE: endometriotic epithelial cells (proliferative phase: n = 10). ENS: endometriotic stromal cells (proliferative phase: n = 10). a: p<.05 versus control (C) cells.
(TIF)

Table S1 **Sequences of the primers used for mRNA quantitation by real-time RT-PCR.**
(DOCX)

Table S2 **Percent inhibition of cell proliferation in endometrial epithelial and stromal cells following treatment with CGP049090 versus PKF 115–854.**
(DOCX)

Table S3 **Percent inhibition of cell proliferation in endometriotic epithelial and stromal cells following treatment with CGP049090 versus PKF 115–854.**
(DOCX)

Table S4 **Survivin mRNA expression in non-treated and PKF 115–584–treated endometrial epithelial and stromal cells of patients with and without endometriosis.**
(DOCX)

Table S5 **MMP-2 mRNA expression in non-treated and PKF 115–584–treated endometrial epithelial and stromal cells of patients with and without endometriosis.**
(DOCX)

Table S6 **MMP-9 mRNA expression in non-treated and PKF 115–584–treated endometrial epithelial and stromal cells of patients with and without endometriosis.**
(DOCX)

Table S7 **c-Myc mRNA expression in non-treated and PKF 115–584–treated endometrial epithelial and stromal cells of patients with and without endometriosis.**
(DOCX)

Table S8 **Hyaluronidase-2 mRNA expression in non-treated and PKF 115–584–treated epithelial and stromal cells of endometriotic tissue and matched eutopic endometrium of the same patients.**
(DOCX)

Table S9 **Survivin mRNA expression in non-treated and PKF 115–584–treated epithelial and stromal cells of endometriotic tissue and matched eutopic endometrium of the same patients.**
(DOCX)

Table S10 **MMP-2 mRNA expression in non-treated and PKF 115–584–treated epithelial and stromal cells of endometriotic tissue and matched eutopic endometrium of the same patients.**
(DOCX)

Table S11 **MMP-9 mRNA expression in non-treated and PKF 115–584–treated epithelial and stromal cells of endometriotic tissue and matched eutopic endometrium of the same patients.**
(DOCX)

Table S12 **c-Myc mRNA expression in non-treated and PKF 115–584–treated epithelial and stromal cells of endometriotic tissue and matched eutopic endometrium of the same patients.**
(DOCX)
