# Supplementary figures and images for: In Vitro Effects of a Small-Molecule Antagonist of the Tcf/ß-Catenin Complex on Endometrial and Endometriotic Cells of Patients with Endometriosis
Source: PLoS One. 2013 Apr 23;8(4):e61690. doi: 10.1371/journal.pone.0061690 (PMC3634014; doi:10.1371/journal.pone.0061690)

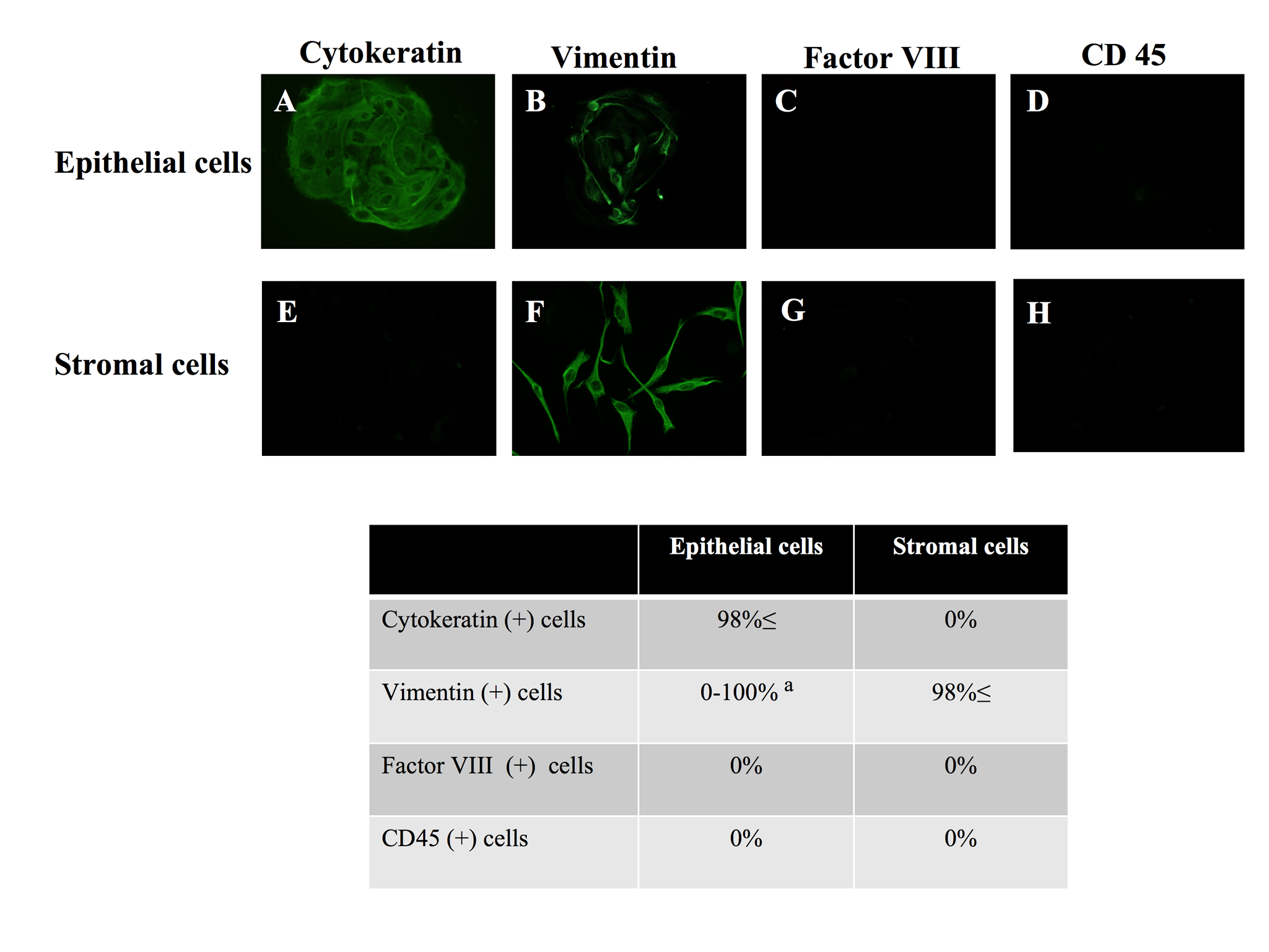

Supplement: Figure S1 — Representative photomicrographs of immunocytochemistry for cytokeratin (A, E), vimentin (B, F), factor VIII (C, G) and CD 45 (D, H) in isolated endometrial epithelial (A–D) and stromal cells (E–H). Original magnification: ×400. Percentage of cytokeratin, vimentin, factor VIII or CD 45 positive epithelial and stromal cells. a: Although vimentin is a mesenchymal marker, it is also expressed in mesoderm-derived epithelium, such as endometrium (3).Vimentin is also expressed in endometriotic epithelial cells (3, 36). (TIFF) [file pone.0061690.s001.tiff]

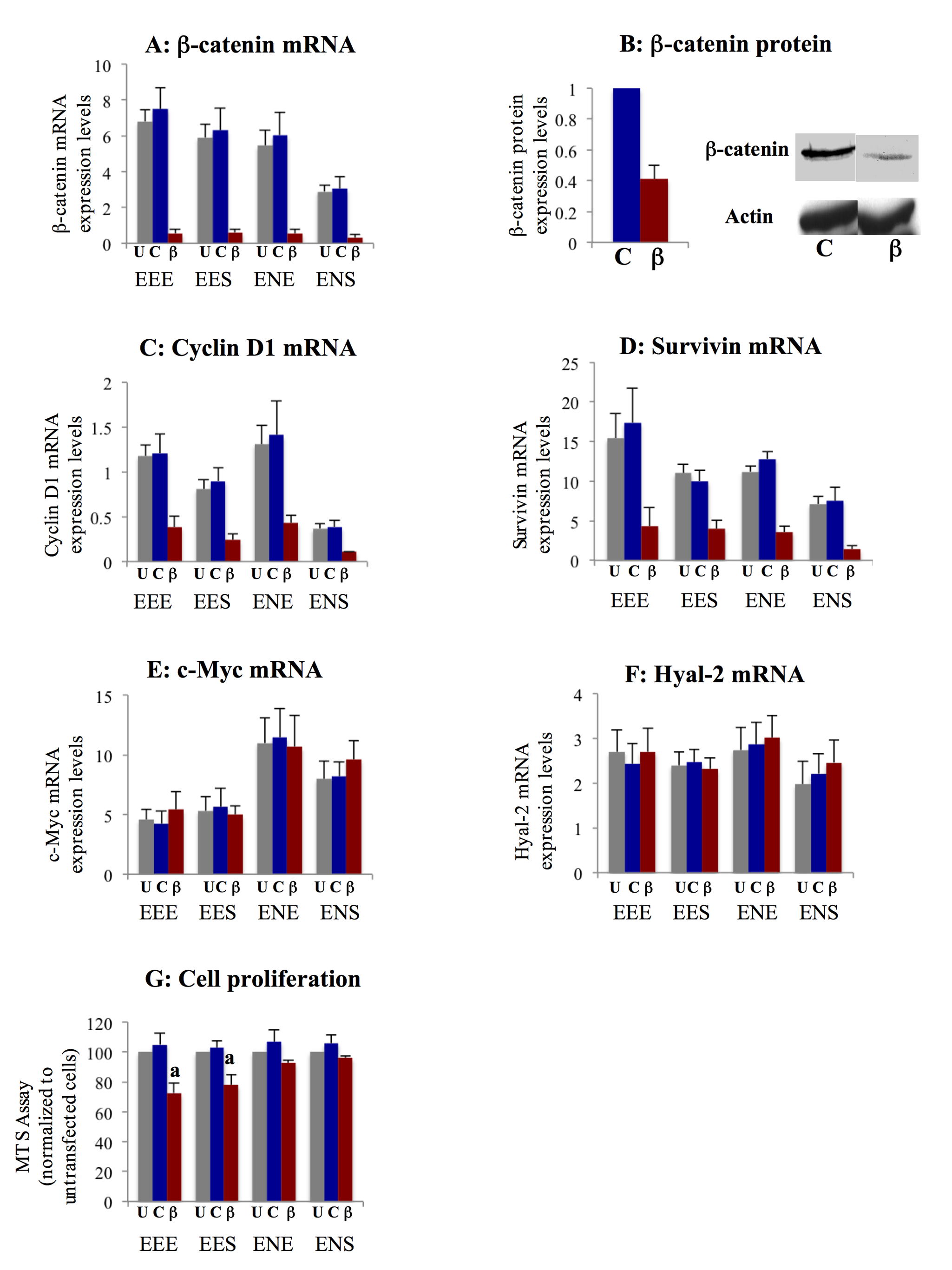

Supplement: Figure S2 — A: ß-catenin mRNA expression in untransfected (U), control (C) or ß-catenin siRNA-transfected (ß) cells. B: Western blot analysis of ß-catenin protein expression in control (C) or ß-catenin siRNA-transfected (ß) endometrial stromal cells (n = 5) and representative photomicrographs of western blot analysis. C: Cyclin D1 mRNA expression in untransfected (U), control (C) or ß-catenin siRNA-transfected (ß) cells. D: Survivin mRNA expression in untransfected (U), control (C) or ß-catenin siRNA-transfected (ß) cells. E: c-Myc mRNA expression in untransfected (U), control (C) or ß-catenin siRNA-transfected (ß) cells. F: Hyaluronidase-2 (Hyal-2) mRNA expression in untransfected (U), control (C) or ß-catenin siRNA-transfected (ß) cells. G: Cell proliferation in untransfected (U), control (C) or ß-catenin siRNA-transfected (ß) cells. Numerical values are presented as the mean+SEM. Expression levels of ß-catenin, Cyclin D1, Survivin, c-Myc mRNA and Hyaluronidase-2 are given relative to the expression levels of the reference gene, GAPDH. ß-catenin protein expression in ß-catenin siRNA-transfected cells (ß) was normalized to respective controls (C). Cell proliferation in control (C) or ß-catenin siRNA-transfected (ß) cells was normalized to untransfected (U) cells. EEE: endometrial epithelial cells of patients with endometriosis (proliferative phase: n = 10). EES: endometrial stromal cells of patients with endometriosis (proliferative phase: n = 10). ENE: endometriotic epithelial cells (proliferative phase: n = 10). ENS: endometriotic stromal cells (proliferative phase: n = 10). a: p<.05 versus control (C) cells. (TIF) [file pone.0061690.s002.tiff]
