# Supplementary material for: In Vitro Effects of a Small-Molecule Antagonist of the Tcf/ß-Catenin Complex on Endometrial and Endometriotic Cells of Patients with Endometriosis
Source: PLoS One. 2013 Apr 23;8(4):e61690. doi: 10.1371/journal.pone.0061690 (PMC3634014; doi:10.1371/journal.pone.0061690)
Supplement: Table S1 — Sequences of the primers used for mRNA quantitation by real-time RT-PCR. (DOCX) [file pone.0061690.s003.docx]

**Table S1: Sequences of the primers used for mRNA quantitation by real-time RT-PCR**

| Gene | Sense primers | Antisense primers |
| --- | --- | --- |
| Cyclin D1 | 5´-GTGGGTGTGCAAGCCAGGT-3´ | 5´-TTCCTGTCCTACTACCGCCT-3´ |
| MMP-2 | 5´-tggcaagtacggcttctgtc-3´ | 5´-ttcttgtcgcggtcgtagtc-3´ |
| MMP-9 | 5´-AGAGATGCGTGGAGAGTCGAAA-3´ | 5´-AAGGTTTGGAATCTGCCCAGGT-3´ |
| ß-catenin | 5'-CGTTTGGCTGAACCATCA-3' | 5'-TGAGGAGAACGCATGATAGCG-3' |
| Survivin | 5´-GGACCACCGCATCTCTACAT-3´ | 5´-GACAGAAAGGAAAGCGCAAC-3´ |
| c-Myc | 5´-TTTTTCGGGTAGTGGAAAACC-3´ | 5´-TTCCTGTTGGTGAAGCTAACG-3´ |
| Hyal-2 | 5´-TGTGAGCTTCCGTGTTCAG-3´ | 5´-GTCTCCGTGCTTGTGGTGTA-3´ |
| GAPDH | 5'-TGCACCACCAACTGCTTAG-3' | 5'-CTCTCGTTCACCTCGATCTTCA-3' |

MMP-2: matrix metalloproteinase-2

MMP-9: matrix metalloproteinase-9

Hyal-2: hyaluronidases 2

GAPDH: glyceraldehyde 3-phosphate dehydrogenase
