# Supplementary material for: In Vitro Effects of a Small-Molecule Antagonist of the Tcf/ß-Catenin Complex on Endometrial and Endometriotic Cells of Patients with Endometriosis
Source: PLoS One. 2013 Apr 23;8(4):e61690. doi: 10.1371/journal.pone.0061690 (PMC3634014; doi:10.1371/journal.pone.0061690)
Supplement: Table S2 — Percent inhibition of cell proliferation in endometrial epithelial and stromal cells following treatment with CGP049090 versus PKF 115–854. (DOCX) [file pone.0061690.s004.docx]

**Table S2: Percent inhibition of cell proliferation in endometrial epithelial and stromal cells following treatment with CGP049090 versus PKF 115-854.**

| Menstrual | Epithelial cells | | Stromal cells | |
| --- | --- | --- | --- | --- |
| cycle |  | |  | |
|  | CGP049090 | PKF 115-584 | CGP049090 | PKF 115-584 |
|  | (6.25 µM) | (6.25 µM) | (6.25 µM) | (6.25 µM) |
| M | 21.5 ± 9.6 % (8) ^a^ | 74.5 ± 4.1 % (8) | 29.1 ± 2.4 % (8) ^a^ | 58.2 ± 9.5 % (8) |
| P | 27.8 ± 8.6 % (14) ^a^ | 72.0 ± 5.6 % (14) | 18.8 ± 9.0 % (14) ^a^ | 64.5 ± 9.3 % (14) |
| ES | 42.0 ± 12.4 % (10) ^a^ | 79.4 ± 4.5 % (10) | 25.6 ± 8.5 % (10) ^a^ | 63.7 ± 12.8 % (10) |
| MS | 23.5 ± 7.8 % (12) ^a^ | 83.4 ± 3.7 % (12) | 19.1 ± 8.7 % (12) ^a^ | 59.8 ± 8.4 % (12) |
| LS | 27.8 ± 14.3 % (6) ^a^ | 59.1 ± 9.1 % (6) | 14.4 ± 10.4 % (6) ^a^ | 43.1 ± 1.6 % (6) |

All data are expressed as mean ± SEM.

Values in parentheses indicate the number of samples examined for effects of PKF 115-854 and CGP049090 on cell proliferation

M: menstrual phase, P: proliferative phase, ES: early secretory phase, MS: mid- secretory phase, LS: late secretory phase

a: p<.01 versus treatment with PKF 115-584
