# Supplementary material for: In Vitro Effects of a Small-Molecule Antagonist of the Tcf/ß-Catenin Complex on Endometrial and Endometriotic Cells of Patients with Endometriosis
Source: PLoS One. 2013 Apr 23;8(4):e61690. doi: 10.1371/journal.pone.0061690 (PMC3634014; doi:10.1371/journal.pone.0061690)
Supplement: Table S3 — Percent inhibition of cell proliferation in endometriotic epithelial and stromal cells following treatment with CGP049090 versus PKF 115–854. (DOCX) [file pone.0061690.s005.docx]

**Table S3: Percent inhibition of cell proliferation in endometriotic epithelial and stromal cells following treatment with CGP049090 versus PKF 115-854.**

| Menstrual | Epithelial cells | | Stromal cells | |
| --- | --- | --- | --- | --- |
| cycle |  | |  | |
|  | CGP049090 | PKF 115-584 | CGP049090 | PKF 115-584 |
|  | (6.25 µM) | (6.25 µM) | (6.25 µM) | (6.25 µM) |
| P | 35.0 ± 9.7 % (14) | 50.2 ± 8.2 % (14) | 27.6 ± 9.8 % (14) | 30.5 ± 9.1 % (14) |
| S | 19.5 ± 7.2 % (13) | 34.6 ± 8.9 % (13) | 27.5 ± 8.3 % (16) | 32.9 ± 10.4 % (16) |

All data are expressed as mean ± SEM.

Values in parentheses indicate the number of samples examined for effects of PKF 115-854 and CGP049090 on cell proliferation

P: proliferative phase, S: secretory phase
