# Supplementary material for: In Vitro Effects of a Small-Molecule Antagonist of the Tcf/ß-Catenin Complex on Endometrial and Endometriotic Cells of Patients with Endometriosis
Source: PLoS One. 2013 Apr 23;8(4):e61690. doi: 10.1371/journal.pone.0061690 (PMC3634014; doi:10.1371/journal.pone.0061690)
Supplement: Table S4 — Survivin mRNA expression in non-treated and PKF 115–584–treated endometrial epithelial and stromal cells of patients with and without endometriosis. (DOCX) [file pone.0061690.s006.docx]

**Table S4: Survivin mRNA expression in non-treated and PKF 115-584–treated endometrial epithelial and stromal cells of patients with and without endometriosis.**

| Endo + | | | | Endo - | | | |
| --- | --- | --- | --- | --- | --- | --- | --- |
| Epithelial cells | | Stromal cells | | Epithelial cells | | Stromal cells | |
| Non-treated | Treated | Non-treated | Treated | Non-treated | Treated | No-treated | Treated |
| 11.4 ± 4.3 | 1.1 ± 0.4 | 10.9 ± 2.1 | 0.9 ± 0.3 | 11.7 ± 3.1 | 0.9 ± 0.3 | 12.5 ± 3.3 | 1.5 ± 0.5 |
| (52) | (52) | (52) | (52) | (52) | (52) | (52) | (52) |

Expression levels of Survivin mRNA are given relative to the expression levels of the reference gene,

GAPDH.

All data are expressed as mean ± SEM.

Values in parentheses indicate the number of samples examined for Survivin mRNA expression.

Endo (+): Endometrium of patients with endometriosis, Endo (-): endometrium of patients without endometriosis
