# Supplementary material for: In Vitro Effects of a Small-Molecule Antagonist of the Tcf/ß-Catenin Complex on Endometrial and Endometriotic Cells of Patients with Endometriosis
Source: PLoS One. 2013 Apr 23;8(4):e61690. doi: 10.1371/journal.pone.0061690 (PMC3634014; doi:10.1371/journal.pone.0061690)
Supplement: Table S5 — MMP-2 mRNA expression in non-treated and PKF 115–584–treated endometrial epithelial and stromal cells of patients with and without endometriosis. (DOCX) [file pone.0061690.s007.docx]

**Table S5: MMP-2 mRNA expression in non-treated and PKF 115-584–treated endometrial epithelial and stromal cells of patients with and without endometriosis.**

| Menstrual | Endo + | | | | Endo - | | | |
| --- | --- | --- | --- | --- | --- | --- | --- | --- |
| cycle |  | | | |  | | | |
|  | Epithelial cells | | Stromal cells | | Epithelial cells | | Stromal cells | |
|  | Non-treated | Treated | Non-treated | Treated | Non-treated | Treated | Non-treated | Treated |
| M | 17.7 ± 13.7 ^a, b^ | 0.7 ± 0.3 | 16.2 ± 12.1 | 0.4 ± 0.2 | 1.5 ± 0.7 | 0.06 ± 0.02 | 1.6 ± 0.8 | 0.7 ± 0.05 |
|  | (6) | (6) | (6) | (6) | (6) | (6) | (6) | (6) |
| P | 0.8 ± 0.3 | 0.07 ± 0.03 | 7.1 ± 1.9 | 0.4± 0.2 | 1.0 ± 0.4 | 0.01 ± 0.008 | 1.6 ± 0.4 | 0.01 ± 0.003 |
|  | (20) | (20) | (20) | (20) | (20) | (20) | (20) | (20) |
| ES | 0.9 ± 0.3 | 0.1 ± 0.05 | 3.4 ± 0.1 | 0.1 ± 0.05 | 0.8 ± 0.1 | 0.01 ± 0.001 | 1.0 ± 0.2 | 0.02 ± 0.001 |
|  | (7) | (7) | (7) | (7) | (7) | (7) | (7) | (7) |
| MS | 1.14 ± 0.7 | 0.04 ± 0.02 | 0.7 ± 0.2 | 0.02 ± 0.005 | 1.05 ± 0.7 | 0.02 ± 0.01 | 0.9 ± 0.3 | 0.051 ± 0.004 |
|  | (15) | (15) | (15) | (15) | (15) | (15) | (15) | (15) |
| LS | 0.6 ± 0.1 | 0.02 ± 0.004 | 0.7 ± 0.1 | 0.02 ± 0.007 | 0.8 ± 0.1 | 0.05 ± 0.005 | 0.8 ± 0.09 | 0.02 ± 0.001 |
|  | (4) | (4) | (4) | (4) | (4) | (4) | (4) | (4) |

Expression levels of MMP-2 mRNA are given relative to the expression levels of the reference gene,

GAPDH.

All data are expressed as mean ± SEM.

Values in parentheses indicate the number of samples examined for MMP-2 mRNA expression.

Endo (+): Endometrium of patients with endometriosis, Endo (-): endometrium of patients without endometriosis

M: menstrual phase, P: proliferative phase, ES: early secretory phase, MS: mid- secretory phase, LS: late secretory phase

a: p<.05 versus non-treated epithelial cells of patients without endometriosis from the menstrual phase

b: p<.05 versus non-treated epithelial cells of patients with endometriosis from the proliferative phase, and the early-, mid- and late-secretory phases.
