# Supplementary material for: In Vitro Effects of a Small-Molecule Antagonist of the Tcf/ß-Catenin Complex on Endometrial and Endometriotic Cells of Patients with Endometriosis
Source: PLoS One. 2013 Apr 23;8(4):e61690. doi: 10.1371/journal.pone.0061690 (PMC3634014; doi:10.1371/journal.pone.0061690)
Supplement: Table S6 — MMP-9 mRNA expression in non-treated and PKF 115–584–treated endometrial epithelial and stromal cells of patients with and without endometriosis. (DOCX) [file pone.0061690.s008.docx]

**Table S6: MMP-9 mRNA expression in non-treated and PKF 115-584–treated endometrial epithelial and stromal cells of patients with and without endometriosis.**

| Menstrual | Endo + | | | | Endo - | | | |
| --- | --- | --- | --- | --- | --- | --- | --- | --- |
| cycle |  | | | |  | | | |
|  | Epithelial cells | | Stromal cells | | Epithelial cells | | Stromal cells | |
|  | Non-treated | Treated | Non-treated | Treated | Non-treated | Treated | No-treated | Treated |
| M | 16.9 ± 9.4 ^a^ | 1.1 ± 0.8 | 10.9 ± 6.4 | 0.4 ± 0.2 | 7.7 ± 5.7 | 0.8 ± 0.08 | 5.84 ± 4.4 | 0.03 ± 0.01 |
|  | (6) | (6) | (6) | (6) | (6) | (6) | (6) | (6) |
| P | 1.4 ± 0.6 | 0.08 ± 0.02 | 2.0 ± 1.3 | 0.09 ± 0.04 | 1.1 ± 0.2 | 0.02 ± 0.001 | 1.1± 0.4 | 0.04 ± 0.02 |
|  | (20) | (20) | (20) | (20) | (20) | (20) | (20) | (20) |
| ES | 1.2 ± 0.6 | 0.07 ± 0.008 | 2.9 ± 1.7 | 0.04 ± 0.02 | 1.3 ± 0.5 | 0.02 ± 0.002 | 1.4 ± 0.9 | 0.01 ± 0.001 |
|  | (7) | (7) | (7) | (7) | (7) | (7) | (7) | (7) |
| MS | 1.0 ± 0.4 | 0.02 ± 0.006 | 1.8 ± 0.6 | 0.02 ± 0.01 | 1.2 ± 0.9 | 0.04 ± 0.01 | 1.5 ± 0.5 | 0.04 ± 0.02 |
|  | (15) | (15) | (15) | (15) | (15) | (15) | (15) | (15) |
| LS | 1.0 ± 0.8 | 0.02 ± 0.002 | 1.1 ± 0.6 | 0.03 ± 0.01 | 0.8 ± 0.2 | 0.02 ± 0.01 | 1.2 ± 0.03 | 0.03 ± 0.001 |
|  | (4) | (4) | (4) | (4) | (4) | (4) | (4) | (4) |

Expression levels of MMP-9 mRNA are given relative to the expression levels of the reference gene,

GAPDH.

All data are expressed as mean ± SEM.

Values in parentheses indicate the number of samples examined for MMP-9 mRNA expression.

Endo (+): Endometrium of patients with endometriosis, Endo (-): endometrium of patients without endometriosis

M: menstrual phase, P: proliferative phase, ES: early secretory phase, MS: mid- secretory phase, LS: late secretory phase

a: p<.05 versus non-treated epithelial cells of patients with endometriosis from the proliferative phase, and the early-, mid- and late-secretory phases.
