# Supplementary material for: In Vitro Effects of a Small-Molecule Antagonist of the Tcf/ß-Catenin Complex on Endometrial and Endometriotic Cells of Patients with Endometriosis
Source: PLoS One. 2013 Apr 23;8(4):e61690. doi: 10.1371/journal.pone.0061690 (PMC3634014; doi:10.1371/journal.pone.0061690)
Supplement: Table S7 — c-Myc mRNA expression in non-treated and PKF 115–584–treated endometrial epithelial and stromal cells of patients with and without endometriosis. (DOCX) [file pone.0061690.s009.docx]

**Table S7: c-Myc mRNA expression in non-treated and PKF 115-584–treated endometrial epithelial and stromal cells of patients with and without endometriosis.**

| Endo + | | | | Endo - | | | |
| --- | --- | --- | --- | --- | --- | --- | --- |
| Epithelial cells | | Stromal cells | | Epithelial cells | | Stromal cells | |
| Non-treated | Treated | Non-treated | Treated | Non-treated | Treated | No-treated | Treated |
| 4.9 ± 0.8 | 5.3 ± 0.9 | 4.4 ± 0.6 | 4.1 ± 0.8 | 3.2 ± 0.7 | 3.8 ± 0.8 | 3.3 ± 0.7 | 3.7 ± 0.8 |
| (52) | (52) | (52) | (52) | (52) | (52) | (52) | (52) |

Expression levels of c-Myc mRNA are given relative to the expression levels of the reference gene, GAPDH.

All data are expressed as mean ± SEM.

Values in parentheses indicate the number of samples examined for c-Myc mRNA expression.

Endo (+): Endometrium of patients with endometriosis, Endo (-): endometrium of patients without endometriosis
