# Supplementary material for: In Vitro Effects of a Small-Molecule Antagonist of the Tcf/ß-Catenin Complex on Endometrial and Endometriotic Cells of Patients with Endometriosis
Source: PLoS One. 2013 Apr 23;8(4):e61690. doi: 10.1371/journal.pone.0061690 (PMC3634014; doi:10.1371/journal.pone.0061690)
Supplement: Table S8 — Hyaluronidase-2 mRNA expression in non-treated and PKF 115–584–treated epithelial and stromal cells of endometriotic tissue and matched eutopic endometrium of the same patients. (DOCX) [file pone.0061690.s010.docx]

**Table S8: Hyaluronidase-2 mRNA expression in non-treated and PKF 115-584–treated epithelial and stromal cells of endometriotic tissue and matched eutopic endometrium of the same patients.**

| Endometriosis | | | | Matched eutopic endometrium | | | |
| --- | --- | --- | --- | --- | --- | --- | --- |
| Epithelial cells | | Stromal cells | | Epithelial cells | | Stromal cells | |
| Non-treated | Treated | Non-treated | Treated | Non-treated | Treated | Non-treated | Treated |
| 2.9 ± 0.8 | 2.2 ± 0.7 | 1.9 ± 0.5 | 2.1 ± 0.5 | 2.4 ± 0.4 | 2.7 ± 0.4 | 2.0 ± 0.4 | 2.1 ± 0.4 |
| (24) | (24) | (24) | (24) | (24) | (24) | (24) | (24) |

Expression levels of Hyaluronidase-2 mRNA are given relative to the expression levels of the reference gene, GAPDH.

All data are expressed as mean ± SEM.

Values in parentheses indicate the number of samples examined for Hyaluronidase-2 mRNA expression.
