# Supplementary material for: In Vitro Effects of a Small-Molecule Antagonist of the Tcf/ß-Catenin Complex on Endometrial and Endometriotic Cells of Patients with Endometriosis
Source: PLoS One. 2013 Apr 23;8(4):e61690. doi: 10.1371/journal.pone.0061690 (PMC3634014; doi:10.1371/journal.pone.0061690)
Supplement: Table S9 — Survivin mRNA expression in non-treated and PKF 115–584–treated epithelial and stromal cells of endometriotic tissue and matched eutopic endometrium of the same patients. (DOCX) [file pone.0061690.s011.docx]

**Table S9: Survivin mRNA expression in non-treated and PKF 115-584–treated epithelial and stromal cells of endometriotic tissue and matched eutopic endometrium of the same patients.**

| Endometriosis | | | | Matched eutopic endometrium | | | |
| --- | --- | --- | --- | --- | --- | --- | --- |
| Epithelial cells | | Stromal cells | | Epithelial cells | | Stromal cells | |
| Non-treated | Treated | Non-treated | Treated | Non-treated | Treated | Non-treated | Treated |
| 10.0 ± 3.6 | 0.3 ± 0.07 | 6.1 ± 1.7 | 0.3 ± 0.1 | 10.1 ± 3.4 | 0.8 ± 0.3 | 11.6 ± 3.8 | 0.1 ± 0.03 |
| (24) | (24) | (24) | (24) | (24) | (24) | (24) | (24) |

Expression levels of Survivin mRNA are given relative to the expression levels of the reference gene, GAPDH.

All data are expressed as mean ± SEM.

Values in parentheses indicate the number of samples examined for Survivin mRNA expression.
