# Supplementary material for: In Vitro Effects of a Small-Molecule Antagonist of the Tcf/ß-Catenin Complex on Endometrial and Endometriotic Cells of Patients with Endometriosis
Source: PLoS One. 2013 Apr 23;8(4):e61690. doi: 10.1371/journal.pone.0061690 (PMC3634014; doi:10.1371/journal.pone.0061690)
Supplement: Table S11 — MMP-9 mRNA expression in non-treated and PKF 115–584–treated epithelial and stromal cells of endometriotic tissue and matched eutopic endometrium of the same patients. (DOCX) [file pone.0061690.s013.docx]

**Table S11: MMP-9 mRNA expression in non-treated and PKF 115-584–treated epithelial and stromal cells of endometriotic tissue and matched eutopic endometrium of the same patients.**

| Mentstrual | Endometriosis | | | | Matched eutopic endometrium | | | |
| --- | --- | --- | --- | --- | --- | --- | --- | --- |
| cycle |  | | | |  | | | |
|  | Epithelial cells | | Stromal cells | | Epithelial cells | | Stromal cells | |
|  | Non-treated | Treated | Non-treated | Treated | Non-treated | Treated | Non-treated | Treated |
| P | 1.9 ± 1.1 | 0.07 ± 0.03 | 5.8 ± 3.0 | 0.6 ± 0.4 | 1.2 ± 0.4 | 0.07 ± 0.02 | 1.7 ± 1.0 | 0.08 ± 0.04 |
|  | (12) | (12) | (12) | (12) | (12) | (12) | (12) | (12) |
| S | 5.1 ± 3.0 | 0.08 ± 0.02 | 2.8 ± 1.4 | 0.01 ± 0.004 | 1.0 ± 0.4 | 0.02 ± 0.008 | 2.1 ± 0.9 | 0.03 ± 0.02 |
|  | (12) | (12) | (12) | (12) | (12) | (12) | (12) | (12) |

Expression levels of MMP-9 mRNA are given relative to the expression levels of the reference gene, GAPDH.

All data are expressed as mean ± SEM.

Values in parentheses indicate the number of samples examined for MMP-9 mRNA expression.

P: proliferative phase, S: secretory phase
